# Structure based classification for bile salt export pump (BSEP) inhibitors using comparative structural modeling of human BSEP

**DOI:** 10.1007/s10822-017-0021-x

**Published:** 2017-05-19

**Authors:** Sankalp Jain, Melanie Grandits, Lars Richter, Gerhard F. Ecker

**Affiliations:** Department of Pharmaceutical Chemistry, University of Vienna, Althanstrasse 14, 1090 Vienna, Austria

**Keywords:** BSEP, Structure-based classification, Drug-induced cholestasis, Inhibiton, Transporters, Classification model

## Abstract

The bile salt export pump (BSEP) actively transports conjugated monovalent bile acids from the hepatocytes into the bile. This facilitates the formation of micelles and promotes digestion and absorption of dietary fat. Inhibition of BSEP leads to decreased bile flow and accumulation of cytotoxic bile salts in the liver. A number of compounds have been identified to interact with BSEP, which results in drug-induced cholestasis or liver injury. Therefore, *in silico* approaches for flagging compounds as potential BSEP inhibitors would be of high value in the early stage of the drug discovery pipeline. Up to now, due to the lack of a high-resolution X-ray structure of BSEP, *in silico* based identification of BSEP inhibitors focused on ligand-based approaches. In this study, we provide a homology model for BSEP, developed using the corrected mouse P-glycoprotein structure (PDB ID: 4M1M). Subsequently, the model was used for docking-based classification of a set of 1212 compounds (405 BSEP inhibitors, 807 non-inhibitors). Using the scoring function ChemScore, a prediction accuracy of 81% on the training set and 73% on two external test sets could be obtained. In addition, the applicability domain of the models was assessed based on Euclidean distance. Further, analysis of the protein–ligand interaction fingerprints revealed certain functional group-amino acid residue interactions that could play a key role for ligand binding. Though ligand-based models, due to their high speed and accuracy, remain the method of choice for classification of BSEP inhibitors, structure-assisted docking models demonstrate reasonably good prediction accuracies while additionally providing information about putative protein–ligand interactions.

**Electronic supplementary material:**

The online version of this article (doi:10.1007/s10822-017-0021-x) contains supplementary material, which is available to authorized users.

## Introduction

Transmembrane transport proteins selectively aid in the translocation of molecules across biological membranes by binding the substrate molecules followed by a conformational change [1]. Members of the ATP-binding cassette (ABC) superfamily facilitate the transport of their solutes by using the energy from hydrolysis of ATP. While some ABC-transporters allow specific passage of inorganic ions, others facilitate ATP-dependent transport of organic compounds including xenotoxins, short peptides, lipids, bile acids, glutathione, and glucuronide conjugates. Therefore, ABC-transporters affect the absorption, distribution, metabolism, excretion and toxicity of numerous pharmacological agents. Genetic variations in the genes that encode these transporters lead to disorders such as cystic fibrosis, cholesterol and bile transport defects, as well as neurological diseases [2].

The bile salt export pump (BSEP, gene ABCB11) is a canalicular-specific exporter predominantly expressed in the cholesterol-rich apical membrane of hepatocytes [3]. BSEP facilitates secretion of bile salts from the liver into the bile canaliculi [4–6]. The main function of bile acids is to promote digestion and absorption of dietary fat via formation of micelles [7]. Apart from this, they are increasingly being shown to have hormonal actions throughout the body [8, 9]. Variations in the ABCB11 gene result in different forms of progressive familial intrahepatic cholestasis (PFIC) [10, 11]. PFIC is characterized by an early onset of cholestasis and eventually leads to liver cirrhosis and failure [12–14].

Inhibition of BSEP can result in accumulation of bile salts in the liver, which is considered to be a primary mechanism leading to drug-induced cholestasis—one of the reasons for drug-induced liver injury (DILI) [15–17]. By inhibiting BSEP, drugs such as bosentan, rifampicin and troglitazone cause intracellular accumulation of bile salts and decreased bile flow [18]. Dysfunction due to suppression of gene expression, disturbed signaling or steric inhibition are other important factors leading to DILI [19]. In its Guideline on the Investigation of Drug Interactions (effective: January 2013), the European Medicines Agency (EMA) indicated that BSEP inhibition assessment should be “preferably investigated”. Additionally, EMA states: “If in vitro studies indicate BSEP inhibition, adequate biochemical monitoring including serum bile salts is recommended during drug development” [20]. Furthermore, studies indicate that a majority of drugs that showed in vitro inhibition of BSEP have led to DILI, suggesting that decreased BSEP inhibition is likely to be associated with reduced risk for DILI [17, 21, 22].

With the increasing knowledge of the importance of ABC-transporter for ADMET, also *in silico* models for predicting ligand-transporter interaction became available [23]. With respect to BSEP, QSAR modeling was applied by Warner et al. [24] in which a support vector machine (SVM) model provided the highest accuracy of 87% in the classification of BSEP inhibitors and non-inhibitors on a dataset of 624 compounds [24]. Our group recently published a classification model based on a set of 670 compounds, which allowed the identification of bromocriptine as a BSEP inhibitor [25]. With first X-ray structures of ABC-transporters being published, also structure-based models became available. Bikadi et al. used SVM to predict P-gp substrate binding modes [26, 27]. Dolghih et al. separated P-gp binders from non-binders by applying induced fit docking into the crystal structure of mouse P-gp using the docking score for classification [28]. High area under the curve (AUC) scores of 0.93 and 0.90, respectively were observed for two independent datasets (126 and 64 compounds, respectively). Also Chan et al. [29] evaluated the prediction capability of docking by using 245 P-gp substrates and non-substrates, but the classes were not clearly separated based on the Glide docking scores.

Klepsch et al. [30] showed that docking of a set of propafenones into a homology model of human P-gp reveals poses consistent with QSAR data, and that this can be exploited for the identification of new P-gp inhibitors [31]. Recently, this was enhanced towards a structure-based classification of almost 2000 compounds [32]. Although the docking-based classification showed significantly lower performance than ligand-based models derived from machine learning, it offers information on the molecular basis of protein ligand interaction.

Up to now, due to the lack of a high-resolution X-ray structure of BSEP, no structure-based studies have been performed for this protein. In the present study, we use comparative modeling [33] to create a protein homology model for BSEP by using the corrected mouse P-glycoprotein structure (PDB ID: 4M1M) as template. Subsequently, we developed structure-based classification models using a dataset comprising 408 compounds (113 inhibitors and 295 non-inhibitors) as training set and two external test sets containing 166 compounds (44 inhibitors and 122 non-inhibitors) and 638 compounds (248 inhibitors and 390 non-inhibitors), respectively.

## Materials and methods

### Dataset

A set of 408 compounds (113 inhibitors and 295 non-inhibitors) from the work of Warner et al. [24] was used as the training set and another set containing 166 compounds (44 inhibitors and 122 non-inhibitors) from Pedersen et al. [34] was used as external test set. Both studies provide in vitro inhibition data on human BSEP. While Warner et al. classified compounds with a mean IC_50_ ≤ 300 μM as BSEP inhibitors, in our study we decided to use a much lower threshold (mean IC_50_ ≤ 10 μM) in order to retain only strong inhibitors. Compounds with mean IC_50_ > 300 μM were considered non-inhibitors, and the remaining compounds were excluded from the dataset. Finally, we have a total of 113 strong inhibitors and 295 non-inhibitors. The Pedersen et al. data set is based on inhibition of bile salt export pump (BSEP)-mediated taurocholate (TA) transport in inverted membrane vesicles. After removal of compounds that overlapped with those in our training set, we had a total of 166 compounds (44 strong inhibitors and 122 non-inhibitors) to be used as external test set. In addition, a dataset provided by AstraZeneca within the framework of the IMI project eTOX (http://www.etoxproject.eu) was used as a second external test set to further evaluate our models. The data was measured in a [3H]-taurocholate transport assay performed in Sf21 membrane vesicles using the protocol as described by Dawson et al. [17] and contains the BSEP inhibitory potencies of 1092 compounds as IC_50_ values. Removing the overlapping compounds from the first two datasets resulted in 638 compounds (248 inhibitors and 390 non-inhibitors). All datasets were standardized using the protocol previously described in Montanari et al. [25] and Pinto et al. [35].

### Homology modeling

For human BSEP (UNIPROT ID: O95342), based on sequence identity and atomic resolution, the corrected mouse P-glycoprotein structure (PDB ID: 4M1M) was selected as the most structurally related template protein. Multiple homology models were constructed using MODELLER 9.13 [36] and the Prime module in Maestro [37, 38]. Energy minimized models were then evaluated using DOPE score [39], and GA341 score [40, 41]. The quality of the stereochemical parameters and the normality of the structures were checked using the PROCHECK program included in the PDBsum analysis [42]. Ramachandran plot [43] and G-factor [44], and finally the Q-score [45, 46] values were evaluated to identify the top ranked homology model.

### Molecular dynamics simulation

Molecular dynamics (MD) simulation was carried out in Gromacs 5.0.4 [47–50] using the GROMOS 54a7 forcefield [51]. The protein was placed inside a rectangular box of size 16 × 16 × 16 nm^3^ including approximately 34,000 simple point charge (SPC) water molecules [52]. Sodium and chloride ions were added to gain a neutral system. Energy minimization was carried out with a maximum force of 1000 kJ/mol/nm using the steepest descent algorithm. After the minimization, a NVT equilibration was performed at a constant temperature of 300 K for 100 ps. Followed by a NPT equilibration step for 1 ns, with the pressure set constant at 1 atm and a constant temperature of 300 K. The production simulation was performed at 300 K for 20 ns. The LINCS algorithm [53] was used to constrain the covalent bonds and PME [54] was used to calculate the electrostatic interactions during the simulation. The stability of the protein structure was evaluated by calculating the secondary structure over the simulation time according to the Kabsch and Sander rules [55] and the root-mean-square fluctuation (rmsf) of active site residues (Fig. S1 in the supplementary material). All graphs were created using the XMGrace tool [56].

### Molecular docking and scoring

In order to avoid any bias in the docking studies, the binding site was defined as the complete TM region, taking 20 Å around the coordinate of the center point to allow subsequent flexible docking studies of a series of BSEP inhibitors. The protein was prepared using Protein Preparation Wizard of the Schrödinger Suite (2015) [57, 58]. During this process, hydrogen atoms were added, and optimal protonation states and ASN/GLN/HIS flips were determined. To assess their correct protonation states, ligands were prepared using the LigPrep module of Schrödinger Suite [58, 59] which produces low-energy 3D structures that can be further used for docking studies. The OPLS_2005 force field was used for the minimization of the structures. Different ionization states were generated by adding or removing protons from the ligand at a target pH of 7.0 ± 2.0 using Epik version 3.1 [60, 61]. Tautomers were generated for each ligand. To generate stereoisomers, the information on chirality from the input file for each ligand was retained as is for the entire calculation. This gave a dataset of 1865 structures (318 inhibitors and 1547 non-inhibitors) for the training set, 2009 structures (858 inhibitors and 1151 non-inhibitors) for the external test set from Pedersen et al. and 1560 structures (668 inhibitors and 892 non-inhibitors) for the external test set from AstraZeneca, which were used for docking with the genetic algorithm-based GOLD suit (version 5.2.0) [62, 63].

All the docking runs were performed in high-throughput mode with GOLD. The fitness functions GoldScore (GS) and ChemScore (CS) were used. GlideXP [64, 65] docking from Maestro was also used in order to compare different scoring functions. Finally, all the poses were rescored using an external scoring function, XScore [66]. To gain deeper insights on the binding modes of BSEP inhibitors and non-inhibitors, the protein–ligand interaction fingerprints (PLIF) of the resultant complexes were retrospectively analyzed.

### Machine learning-based model building

The open source software WEKA (version 3.7.10) [67] was used for building binary classification models. The machine learning classifiers: J48, Random Forest, REPTree, LibSVM and Naive Bayes were used with the default parameters along with tenfold internal cross-validation.

### Network-based representation of the dataset

Tanimoto (Tc) similarities between the inhibitors and non-inhibitors of the training set were calculated using MACCS fingerprints [68]. A chemical space network (CSN) [69, 70] was constructed and analyzed in order to assess the structural similarity shared by the compounds of both groups. To show connections between the compounds, a threshold value of 0.7 was set based on the average of Tanimoto max-similarity in the dataset.

### Functional group analysis

Functional group analysis was performed in two stages. First, the substructure patterns of 100 functional groups in SMARTS notation were extracted from the Daylight website (http://www.daylight.com/dayhtml_tutorials/languages/smarts/smarts_examples.html#GROUP). Next, the pattern matching was performed using the SMARTSQueryTool implemented in the Chemistry Development Kit (CDK) [71]. For each functional group, the occurrences of the fragments in a given set of molecules were calculated.

### Protein ligand interaction fingerprints (PLIF)

A PLIF summarizes the interactions between a ligand and a protein using a fingerprint scheme. Here we generated three types of PLIFs that differ in the information encoded. In the first approach, the PLIF encodes the residues involved in an interaction with the ligand in each bit. The second one encodes not only the residue but also the nature of the interaction (e.g. hydrogen bond donor) with the ligand. The third category encodes the functional group of the ligand that interacts with the residue. All the PLIF bits were calculated with the MOE [72] built-in function CalculateRawInteractions using a 1% threshold for molecular interactions and a 20% threshold for surface contacts. The function was embedded in an SVL in-house script and was post processed to enable to calculate functional group PLIFs.

### Applicability domain assessment

An applicability domain (AD) analysis was performed to evaluate if the chemical space covered by the training set used for developing the model is applicable to predict the outcomes of the test sets used to evaluate the model performance. Therefore, AD could provide a first hint if a new chemical structure is covered within the chemical structures or descriptor space of the training set. Many approaches were proposed to estimate AD, for instance based on descriptor ranges, Euclidean distance or probability density, each having their pros and cons. In this study, we implemented the Euclidean distance approach using the KNIME [73] node APD [74, 75] to evaluate if the test sets are within the AD of the training set.

### Performance evaluation

In order to evaluate the quality of our classification models based on the docking studies, we used standard parameters such as count of true positives (TP), false positives (FP), true negatives (TN) and false negatives (FN). Sensitivity (Eq. 1), specificity (Eq. 2) and accuracy (Eq. 3) values were calculated for each model based on the aforementioned parameters to estimate its performance in classifying inhibitors and non-inhibitors. To measure the overall quality of the model, the G-mean (Eq. 4), which takes into account both sensitivity and specificity, and the Matthews’s correlation coefficient (MCC, Eq. 5) were also calculated.

1 $$Sensitivity=~\frac{{TP}}{{\left( {TP+FN} \right)}}$$

2 $$Specificity=~\frac{{TN}}{{\left( {TN+FP} \right)}}$$

3 $$Accuracy=\frac{{\left( {TP+TN} \right)}}{{\left( {TP+FP+TN+FN} \right)}}$$

4 $$G{-}mean=\sqrt {Sensitivity \times Specificity}$$

5 $$MCC = \frac{{\left\{ {\left( {TP \times TN} \right) - \left( {FP \times FN} \right)} \right\}}}{{\left\{ {\left( {TP + FP} \right) \times \left( {TP + FN} \right) \times \left( {TN + FP} \right) \times \left( {TN + FN} \right)} \right\}^{{1/2}} }}$$

### Calculating the probability of prediction

We examined the distribution of docking scores [Chemscore, Goldscore, GlideXP, Xscore (Chemscore) and Xscore (Goldscore)] for the training set molecules. Based on the minimum and maximum score values, the scores were binned in different intervals. Each bin is characterized by the corresponding number of inhibitors and non-inhibitors. Based on these values, we calculated the probability for a molecule to be an inhibitor or a non-inhibitor. A p value (Chi square test) is calculated for each bin to identify the best scoring range that can be used to separate inhibitors from non-inhibitors.

## Results and discussion

### Chemical space network of the dataset

**Fig. 1:**
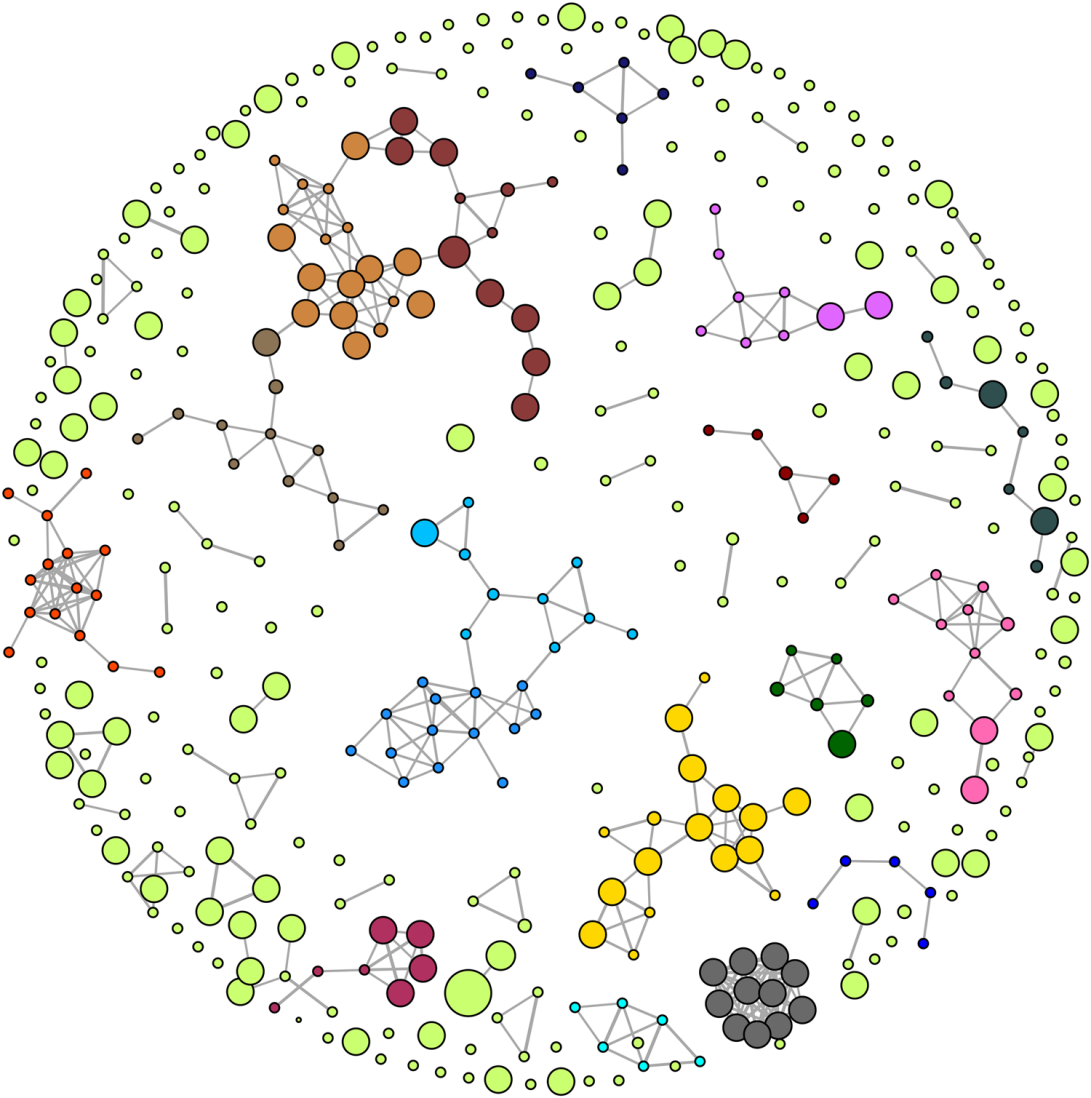
CNS representation of the training set compounds based on MACCS Tc similarity threshold of 0.70. Communities with at least five representative members are color coded

Figure 1 shows the CSN with well-resolved community structures for a set of inhibitors and non-inhibitors from the training set. The representative compounds of some communities are shown in Fig. S2 in the supplementary material. Major community structures [69] (communities with at least five representative members) were algorithmically detected and are color-coded. For our CSN designs, the Fruchterman–Reingold algorithm [76] was applied. The node size is proportional to the activity value (pIC_50_) i.e. the more active the compound, the bigger the node size and vice versa.

A majority of the nodes do not have a connection indicating a high structural diversity in the training dataset. The test dataset from Pedersen et al., showed only three clusters in the CSN with at least five representative members (Fig. S3 in the supplementary material).

### Homology modeling

**Fig. 2:**
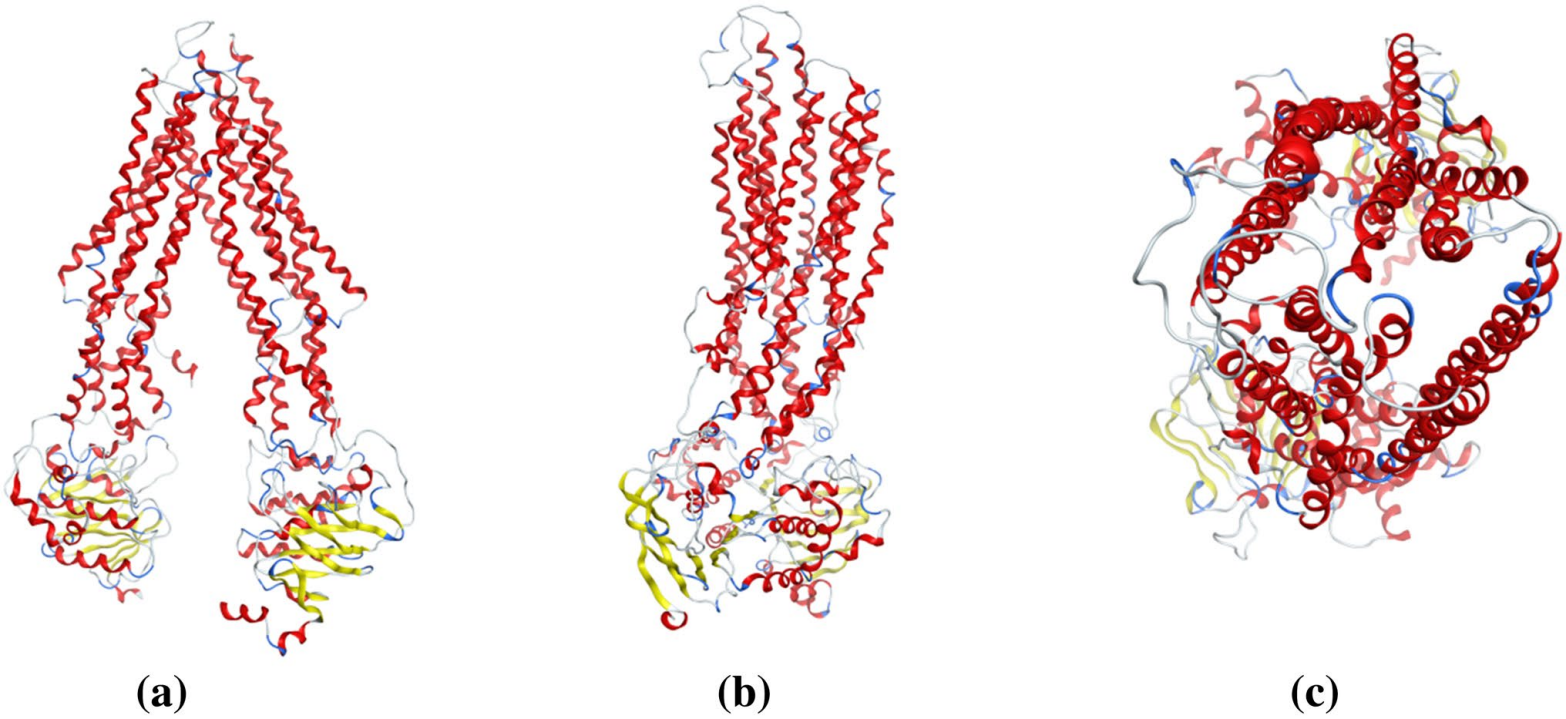
Homology model structure of human BSEP in the inward-facing state. **a** Front view of the transporter. **b** Side view after a 90° rotation. **c** Top view from the extracellular space

**Fig. 3:**
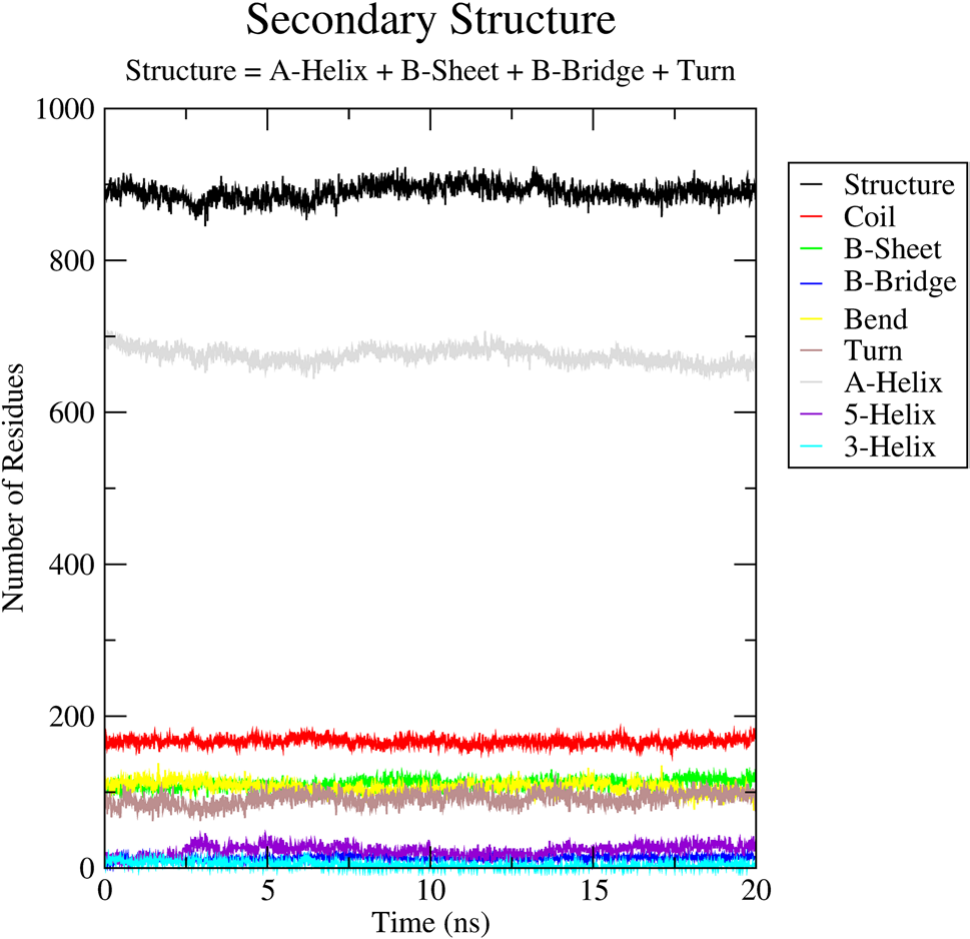
Secondary structure of the protein over the simulation time

Applying the Prime module from Maestro (Schrödinger, Inc. V-10.1.013), a set of homology models of BSEP were created and refined, using the refined mouse P-gp structure as template (PDB ID: 4M1M). The sequence alignment was done using Prime’s alignment program STAin maestro [37, 38] (Fig. S4 in the supplementary material). Analyzing the models with the structure assessment program PROCHECK [42], the best model had a normalized Dope score of −0.625, G-factor −0.12, and Qmean score of 0.597. Furthermore, the Ramachandran plot (Fig. S5 in the supplementary material) showed excellent results, with only 1.9% of residues in generously allowed or disallowed regions. These were all located in the nucleotide binding domains (NBD) or extracellular loops (ECL), and are therefore not involved in drug binding (Fig. S6 in the supplementary material). Based on the study by Mochizuki et al., Asn109, Asn116, Asn122, and Asn125 are residues predicted to be potential glycosylation sites in the extracellular loop (No.1) (EL No.1) of human BSEP [77]. In our final BSEP homology model (Fig. 2), these residues were also found in EL No.1, thus occurring in the correct region of the transmembrane domain (TMD, Fig. S7 in the supplementary material). For further validation, the best model based on normalized Dope score and Qmean score was subject to molecular dynamics simulations for 20 ns. Both the secondary structure of the protein (Fig. 3) as well as the root mean square fluctuation (RMSF < 0.25 nm) of active site residues showed the stability of the structure.

### Docking (structure-based classification)

**Fig. 4:**
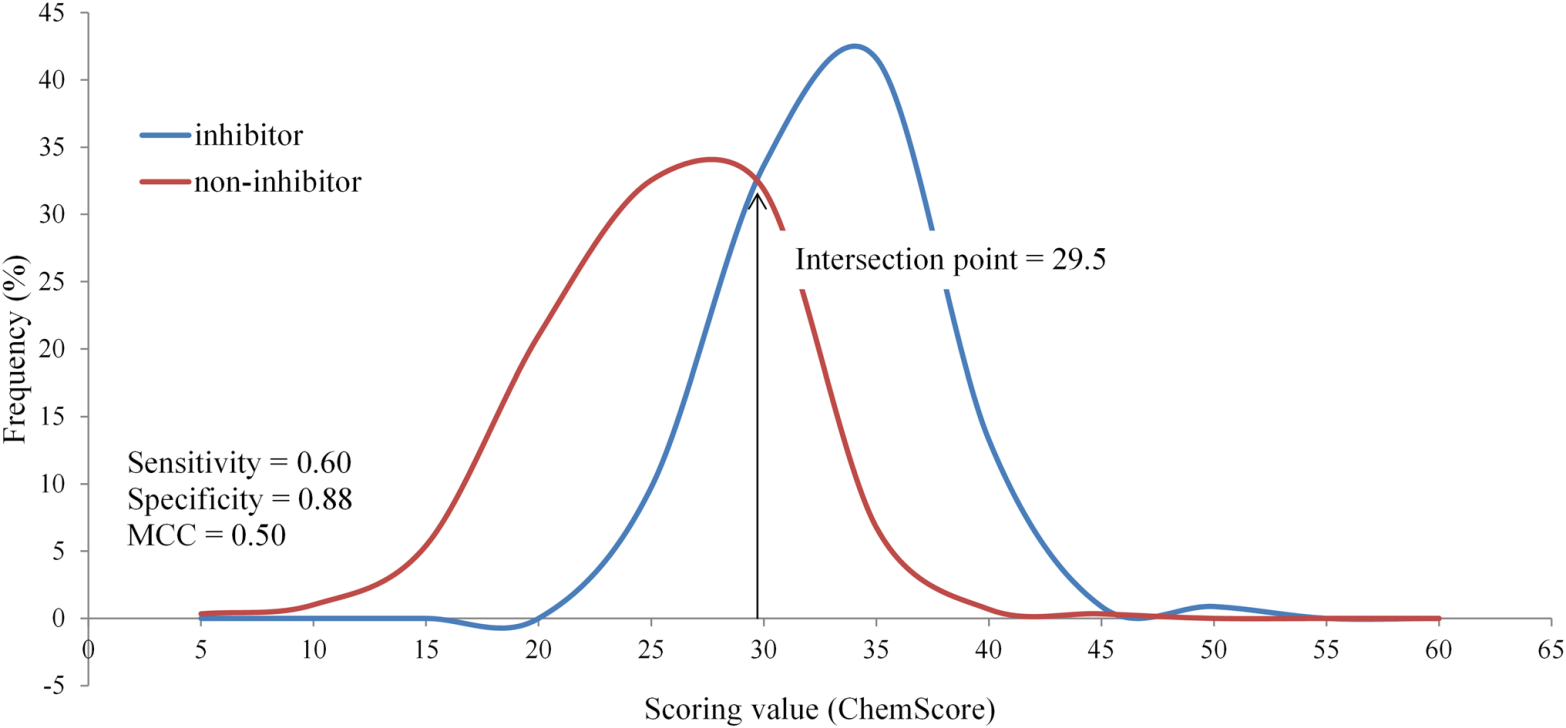
Distribution of BSEP inhibitors and non-inhibitors (training set) based on ChemScore scoring. Sensitivity, specificity, precision and MCC were calculated from the confusion matrix based on the intersection point of both curves

We recently could demonstrate that a validated homology model of P-glycoprotein allowed docking-based classification of inhibitors and non-inhibitors with reasonable performance [32]. Thus, in this study we extended this approach also to BSEP, using a set of 408 compounds (113 inhibitors and 295 non-inhibitors) published by Warner et al. [24] as training set and two data sets as external test set (see “Materials and methods” section). The scores obtained from different fitness functions were binned and the intersection point of the curves for inhibitors and non-inhibitors in the training set served as classification criterion (Fig. 4). Respective confusion matrix parameters and other performance measures are summarized in Table 1. The ChemScore docking run using Xscore as rescoring function retrieved the best performing model with AUC (0.918) and MCC (0.689) measures comparable to the models developed by Warner et al. [24] and Montanari et al. [25]. This model accurately predicted 88% of the training set compounds and 72% of the external test set compounds derived from Pedersen et al. [34] as well as 77% of a set of AstraZeneca internal compounds. The area under the ROC curve (AUC) measure, being independent from class distribution [78, 79], is a good metric for evaluating performance of virtual screening approaches. High AUC values (above 0.8) were observed, indicating a high capacity of the model in ranking compounds by their probability of being inhibitors of BSEP (Figs. S8–S12 in the supplementary material). The results from the AD assessment also show that all compounds from both test sets were found to be within the chemical domain of the training compounds (Table S1 in the supplementary material). Interestingly, the accuracy of predictions did not improve when a consensus of different scoring functions was used.

**Table 1:** Models obtained from different scoring functions based on the training set

Scoring function	Intersection point	AUC	Sensitivity	Specificity	Accuracy	G-mean	MCC
ChemScore	29.50	0.87	0.60	0.88	0.81	0.73	0.50
GoldScore	53.50	0.82	0.74	0.75	0.75	0.74	0.45
GlideXP	−6.80	0.77	0.80	0.65	0.69	0.72	0.39
Xscore (ChemScore)	6.15	0.92	0.71	0.95	0.88	0.82	0.69
Xscore (GoldScore)	6.10	0.93	0.68	0.95	0.88	0.80	0.68

The scoring function in brackets were used to generate the docking poses

### Probability of prediction

For the training set using ChemScore scoring, bin 35–40 gave the maximum number of inhibitors. 88% of inhibitors and 12% of non-inhibitors had the docking score in this range with a p value of 5.9 × 10^−8^. For both test sets, at least 75% of the inhibitors were found to be in this range. Results for different scoring functions can be found in the Table S2 in the supplementary material. Also with the rescoring of ChemScore using Xscore, a particular range could be defined which significantly distinguishes between inhibitors and non-inhibitors. However, this is not the case for GoldScore scoring. With this scoring function no particular docking score range could be identified for the three sets (training set, both test sets) to differentiate between the two classes of compounds with a significant p value. Similar results were obtained using the GlideXP scoring function.

### Analysis of protein ligand interactions

**Fig. 5:**
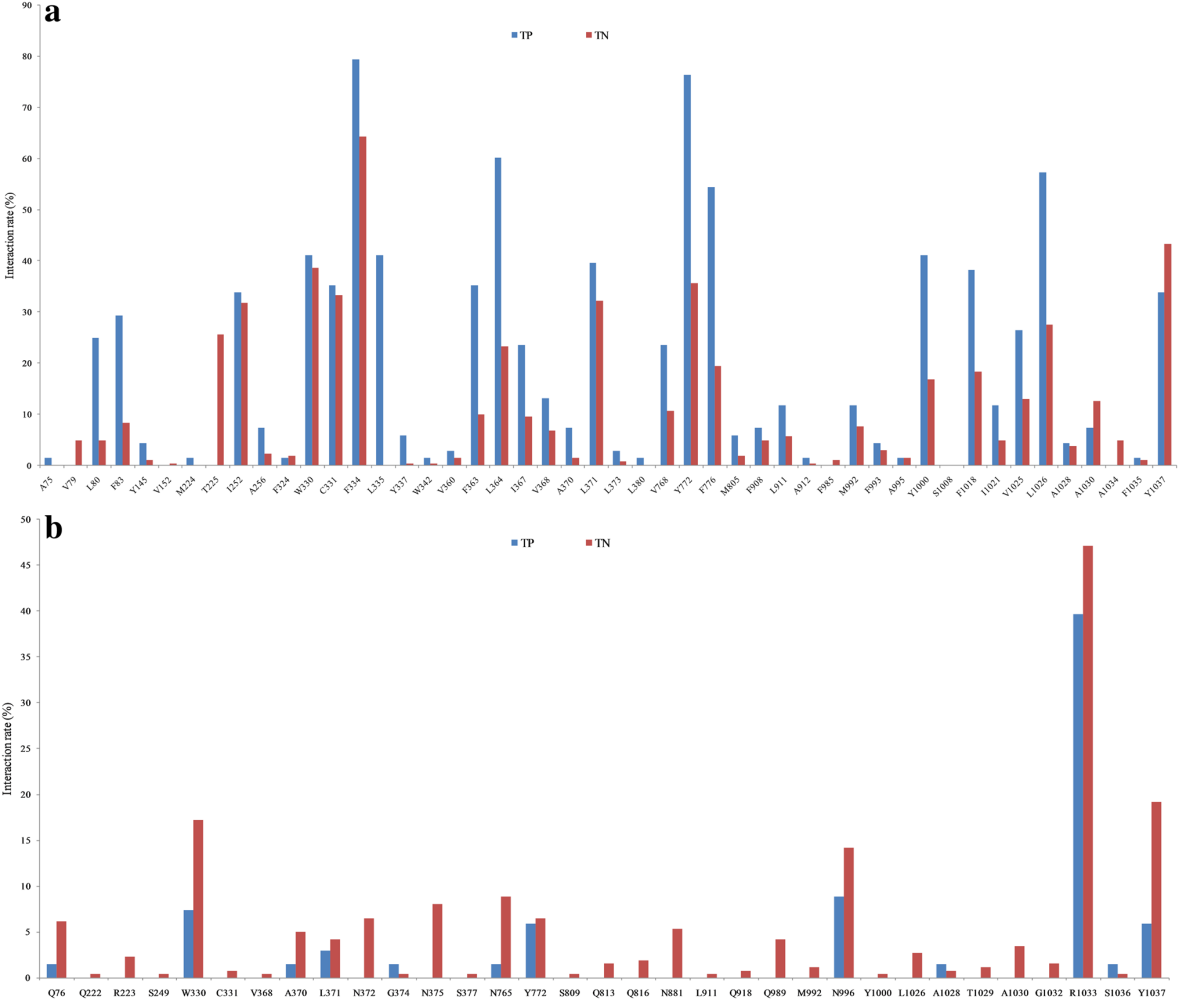
**a** Hydrophobic interaction, **b** hydrogen bond interaction fingerprints of true positives (TP) and true negatives (TN) of the training set. The classification of the compounds is based on the ChemScore scoring function

The Maestro tool allows the computation of different molecular interactions between binding site residues and the corresponding ligand conformation. In this study, the receptor–ligand interaction fingerprint analysis was performed both for the true positives (TPs) and for the true negatives (TNs) on the basis of the docking poses generated. For the training set (Fig. 5) and the two external test sets (Figs. S13, S14 in the supplementary material), the inhibitors showed significantly more hydrophobic interactions with Phe334, Leu364, Tyr772, Phe776 and Leu1026 than non-inhibitors. More than 75% of the inhibitors in the training set and the external test sets showed hydrophobic interactions with Phe334 and Tyr772 (Fig. 5a). In contrast, non-inhibitors showed a higher number of hydrogen bond interactions than inhibitors (Fig. 5b), which points towards the fact that non-inhibitors are more hydrophilic.

**Fig. 6:**
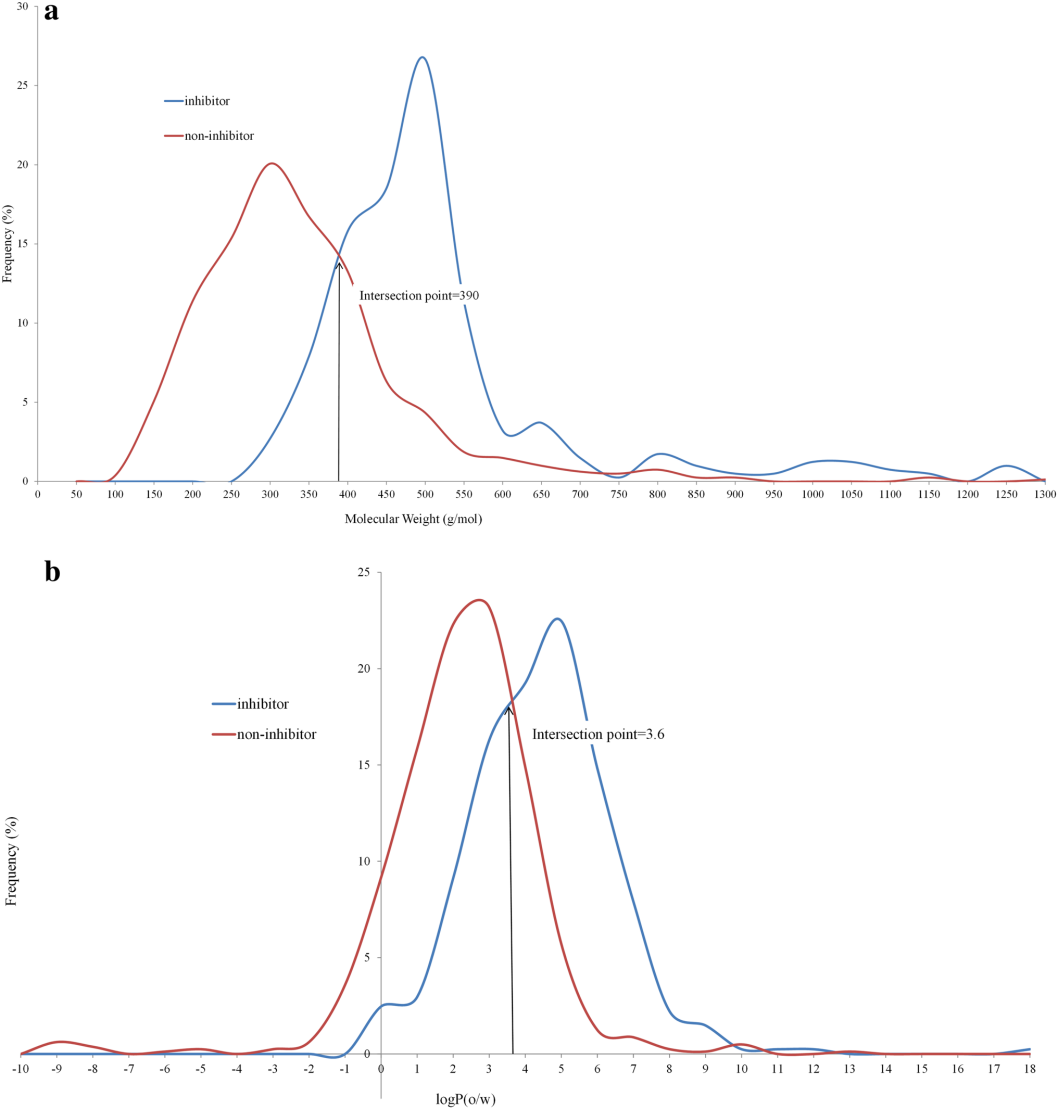
Distribution of BSEP inhibitors and non-inhibitors based on the **a** molecular weight, **b** logP(o/w) of the training set

The significant contribution of hydrophobic interactions prompted us to assess the importance of simple molecular descriptors such as logP and molecular weight. Figure 6 represents the distribution of molecular weight and logP(o/w), respectively, for the training set compounds. Similar distributions, represented in Fig. S15 in the supplementary material, were observed with the external test sets from Pedersen et al. [34] and from AstraZeneca (Fig. S16 in the supplementary material). As proposed by Warner et al. [24], molecular properties such as molecular weight (MW) and logP(o/w) could separate the groups quite well (Table 2). At the intersection of MW = 390 and logP(o/w) = 3.6, 79 and 77% of the compounds were classified correctly. Accordingly, compounds with a molecular weight of 390 or higher or a logP of 3.6 or higher were considered as inhibitors while others were considered as non-inhibitors.

**Table 2:** Models based on physicochemical properties

Molecular property	Intersection point	Sensitivity	Specificity	Accuracy	G-mean	MCC
Molecular weight	390	0.76	0.80	0.79	0.78	0.54
logP	3.6	0.57	0.87	0.77	0.71	0.47

The models based on docking scores (ChemScore and XScore) in combination with molecular weight and logP(o/w) (each normalized) outperformed the other models in terms of MCC and precision. ChemScore and XScore based models, when combined with the physicochemical properties [molecular weight and logP(o/w)] correctly predicted 87 and 88% of training set compounds, giving a MCC value of 0.673 and 0.701 respectively. These models also showed high accuracies as compared to other models for the two external test sets. Detailed accuracy measures are presented in Table S3 in the supplementary material.

Also when poses, generated with GoldScore scoring function and rescored with XScore, were combined with the normalized molecular weight and logP(o/w), it provided accuracies comparable to the former models (Table S3 in the supplementary material). This indicates that considering physicochemical properties of molecules that influence their activity significantly improves the performance of structure-based prediction models.

Distribution of BSEP inhibitors and non-inhibitors using different scoring functions and in combination with physicochemical properties (molecular weight, logP) are presented in Figs. S17–S32 in the supplementary material. A single intersection point could not be obtained, when the rescoring using Xscore (pose generated with GoldScore) was combined with logP(o/w) and thus was not used for the classification of inhibitors and non-inhibitors (Fig. S31 in the supplementary material).

Using the best performing docking scores (ChemScore, XScore) and the descriptors (molecular weight and logP(o/w)) as parameters, we additionally developed machine-learning based binary classification models using J48, Random Forest, REPTree, LibSVMand Naive Bayes in WEKA [67]. These models performed well with accuracies and MCC values (Table S4 in the supplementary material) comparable to those from machine-learning based classification models of Warner et al. [24] and our models previously developed [25].

### Analysis of functional groups and protein–ligand interactions

**Fig. 7:**
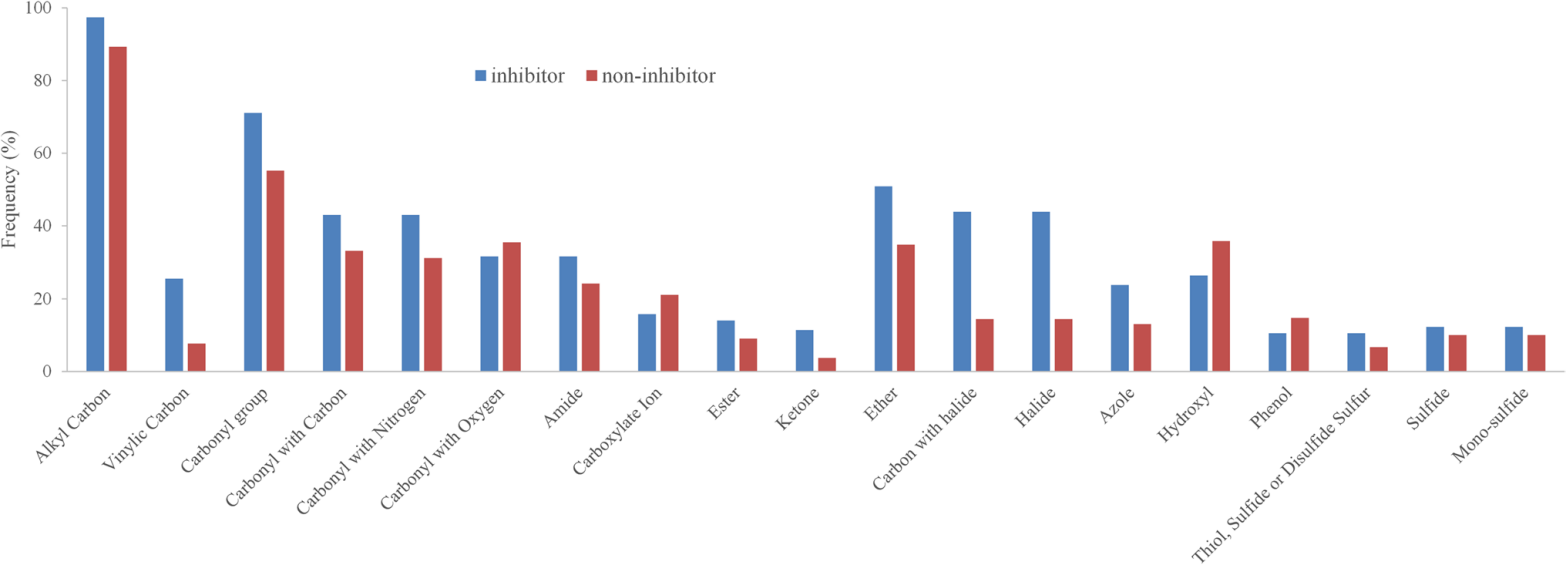
Distribution of functional groups in the training dataset

Next, we investigated the distribution of functional groups between inhibitors and non-inhibitors to identify structural features that are responsible for differences in the activity (inhibitor vs. non-inhibitor). About 70 SMARTS patterns representing the most common functional groups were extracted from the Daylight website (http://www.daylight.com/dayhtml_tutorials/languages/smarts/smarts_examples.html). Basically, groups such as halide/halogen, ether, carbonyl, vinyl carbons (sp2 hybridized) and amide were more frequently found in the inhibitors compared to the non-inhibitors (Fig. 7, S33 in the supplementary material). This further points towards more hydrophobic-driven interactions for inhibitors.

**Fig. 8:**
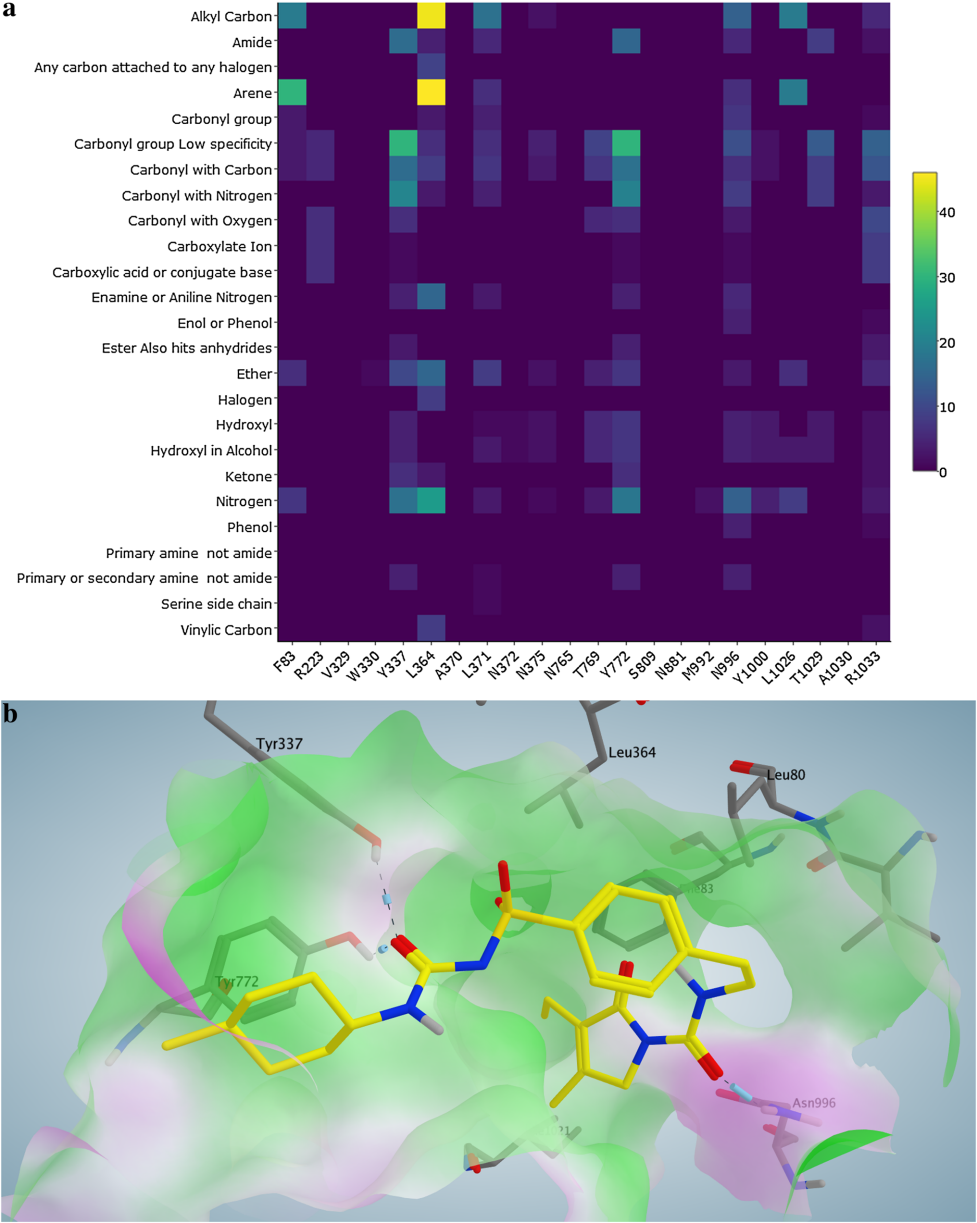
**a** Heat map illustrating the PLIF analysis of the training set inhibitors (*x-axis* contact residues; *y-axis* functional groups of the ligand showing an interaction with the residue; *color scale* number of interacting ligands). **b** Docking pose of Glimepiride (*yellow*) in which its carbonyl groups interact with the residues Tyr337, Tyr772 and Asn996

In addition, we also identified the most frequently occurring interactions between residues and functional groups for the training set compounds. A heat map (Fig. 8a) was generated to illustrate the outcomes of PLIF analysis by displaying the contact residues against the functional groups of the interacting ligands. The color scale represents the amount of ligands which are involved in interactions. Therefore, the most significant interactions between a specific residue and a specific functional group could be visually detected.

We found that the interactions of arene and carbonyl functional groups with tyrosine and leucine are more prominently found among the inhibitors in comparison to the non-inhibitors. We furthered with retrospective assessment of the docking results to check the presence of the aforementioned interactions and evaluated the chances to prioritize a compound as a BSEP inhibitor. Figure 8b represents the docking pose of Glimepiride (yellow) in which its carbonyl groups interact with the residues Tyr337, Tyr772 and Asn996. The residue Leu364 shows a hydrophobic interaction with the arene moiety of the ligand. Similarly, the functional group-residue interactions were confirmed to be present in the docking results of both external test datasets (Figs. S34–S36 in the supplementary material).

Although the functional groups analysis suggests that halide/halogen, carbonyl, ether, vinyl and amide groups were significantly over represented in the inhibitors, only carbonyl group, amide were found to frequently interact with the protein. According to the heat map (Fig. 8a), halide/halogen and vinyl groups do not appear to have a significant number of contacts with the residues. At the same time, arene was found at a similar rate in inhibitors (nearly 95%) and non-inhibitors (nearly 85%), but the PLIF analysis revealed that the arene moiety participates in a significant number of interactions with residues such as Leu364 and Leu1026. This indicates that significant differences in the functional group composition between inhibitors and non-inhibitors (Fig. 7) does not necessarily indicate or provide an outlook on the nature of interactions. This would rather depend on the position of these functional groups in the molecular structure, nature of the binding site residues as well as the size of the binding pocket.

Finally, preliminary results show that the PLIF can also be used as predictor for inhibitor/non inhibitor properties by calculating the Tanimoto distance to known inhibitors. A more detailed description of this approach can be found in the supplementary material.

### Analysis of misclassified compounds

Nearly 90 compounds, altogether from different datasets, were incorrectly classified by all the four scoring functions used in the study. More than 59% of the training set compounds and 48% of the test set compounds were correctly classified by all the scoring functions. Of the 19 misclassified compounds from the training set, nine were predicted as inhibitors and ten were predicted as non-inhibitors.

The training set compound Ebselen was wrongly predicted as non-inhibitor by all scoring functions. Examining its molecular properties revealed that both molecular weight (274) and logP(2.74) fall in the range of non-inhibitors (Table 2). Moreover, the structure of Ebselen was found to be structurally more similar to a set of non-inhibitors compared to the set of inhibitors. Benzylpenicillin (Penicillin G) also belongs to the property space of non-inhibitors (molecular weight = 333.38 and logP = 1.74). Interestingly, both Ebselen and Benzylpenicillin are strong inhibitors (IC_50_< 10 μM) [24]. On the other hand, Phytomenadione (molecular weight = 450.70, logP = 9.05), despite being a non-inhibitor (IC_50_ Y > 1000), was always misclassified as inhibitor. Similar trend was noticed in both external test sets. In total, six inhibitors and 13 non-inhibitors were misclassified from the Pedersen et al. [34] dataset. Interestingly, all six inhibitors were found to be strongly hydrophobic and the molecular properties of about 80% of the non-inhibitors fall in the range of inhibitors. This strengthens the inclusion of this physicochemical properties into the classification model.

### Combining ligand- and structure-based classification (sequential modeling)

Although the structure-based models performed reasonably well, ligand-based methods are considerably faster and perform equally well. Thus, we evaluated if a sequential approach that starts with a ligand-based method and proceeds with screening the positives using structure-based models would improve the precision and reduce the false positives. Therefore, we used an external test set containing 39 inhibitors and 113 non-inhibitors as a starting point. After applying ligand-based classification using the workflow from Montanari et al. [25], 30 inhibitors were correctly predicted (TPs) and there were nine FPs, which leads to a precision of 0.77. After application of our structure-based model based on ChemScore and rescoring using XScore, the precision improved to 0.83, reducing the number of FPs to 5. Further performance measures on the sequential approach are provided in Table 3. Thus, combining ligand- and structure-based models in a sequential setting increased the precision and reduced the calculation time. This might be a versatile approach to reduce the number of FPs when performing large scale *in silico* screening.

**Table 3:** Ligand-based and structure-based classification

Model type	TP	TN	FP	FN	Sensitivity	Specificity	Accuracy	MCC	Precision
**LBC**	**30**	**104**	**9**	**9**	**0.77**	**0.92**	**0.88**	**0.69**	**0.77**
SBC_C	27	91	22	12	0.69	0.81	0.78	0.47	0.55
SBC_G	26	79	34	13	0.67	0.70	0.69	0.33	0.43
SBC_C_X	27	96	17	12	0.69	0.85	0.81	0.52	0.61
LBC + SBC_C	24	107	6	15	0.62	0.95	0.86	0.62	0.80
**LBC + SBC_C_X**	**25**	**108**	**5**	**14**	**0.64**	**0.96**	**0.88**	**0.66**	**0.83**
Consensus	27	106	7	12	0.69	0.94	0.88	0.66	0.79

The best model of the combined approach is highlighted in bold as well as the ligand-based classification

*TP* true positives, *TN* true negatives, *FP* false positives, *FN* false negatives, *LBC* Ligand-based classification (Montanari et al. [25]), *SBC_C* Structure-based classification using ChemScore scoring function, *SBC_G* Structure-based classification using GoldScore scoring function, *SBC_C_X* Structure-based classification using ChemScore scoring function (rescoring using Xscore). Consensus Combination of LBC, SBC_C and SBC_C_X

## Conclusion

Development of structure-based methods for transmembrane transporters of the ABC-family has been less pronounced due to limited availability of experimentally determined 3D structures. However, recent efforts that used homology models of P-glycoprotein provide promising evidences that structure-based classification methods can be applied to these highly flexible and promiscuous proteins. In this study, we used comparative modeling to generate a homology model for the ABC-transporter BSEP and developed structure-based models to classify inhibitors and non-inhibitors. Including logP and molecular weight as an additional layer of information besides the scoring function further increased the performance of the models. PLIF analysis revealed certain functional group-residue interactions that could help to understand the molecular basis of inhibition of the transporter protein by a wide range of ligands. Applicability domain of the models was assessed using Euclidean distance. Furthermore, we estimated the probability of prediction by employing a binning scheme and identified a docking score range that can distinguish a majority of inhibitors from non-inhibitors with high confidence. Finally, combining the structure-based model with our previously published ligand-based classification model in a sequential order provided additional improvement.

Combining ligand- and structure-based models to enhance the performance of virtual screening is of course not a new approach. For receptors and enzymes identification of new ligands quite often starts with a pharmacophore-based screening followed by docking of the top-ranked hits to further refine the shopping list [80]. However, in case of ABC-transporters such as P-glycoprotein, which shows a pronounced polyspecificity in its ligand profile, there is a broad variety of pharmacophore models available. This would render a sequential approach quite challenging. Furthermore, due to the eminent role of ABC-transporters like P-gp, BSEP, and the breast cancer protein (BCRP) in ADME and toxicity, the focus for *in silico* screening lays more on flagging potentially toxic compounds rather than on the identification of new inhibitors for further development as drug candidates. In this setting, machine learning-based classification models might be a better tool for a first computational pre-screening. Therefore, a workflow comprising of prescreening with simple descriptors, classification by machine learning techniques and post processing by structure-based methods might be the workflow of choice to provide accurate prediction combined with additional information on the molecular basis of compound-transporter interaction.

## Electronic supplementary material

Below is the link to the electronic supplementary material.

Supplementary Material 1 (DOCX 2267 kb)
