# Supplementary material for: Structure based classification for bile salt export pump (BSEP) inhibitors using comparative structural modeling of human BSEP
Source: J Comput Aided Mol Des. 2017 May 19;31(6):507–21. doi: 10.1007/s10822-017-0021-x (PMC5487762; doi:10.1007/s10822-017-0021-x)
Supplement: Supplementary file 1 — Supplementary Material 1 (DOCX 2267 kb) [file 10822_2017_21_MOESM1_ESM.docx]

**Supplementary Material**

**Structure Based Classification for Bile Salt Export Pump (BSEP) Inhibitors using Comparative Structural Modeling of Human BSEP**

Sankalp Jain^a^, Melanie Grandits^a^, Lars Richter^a^, Gerhard F. Ecker^a^

*^a^University of Vienna, Department of Pharmaceutical Chemistry, Althanstrasse 14, 1090 Vienna, Austria*

E-Mail: gerhard.f.ecker@univie.ac.at; Phone: +43-1-4277-55110; eFax: +43-1-4277-855110

**Tables**

**Table S1.** Summary view of the applicability domain (AD) analysis with information about the percentage of the reliable and the unreliable predictions

| **Test set (Pedersen et al.)** | | | |
| --- | --- | --- | --- |
| **Scoring function** | **Reliable percentage (%)** | **Unreliable percentage (%)** | **AD limit (Threshold)** |
| Chemscore | 100 (166/166) | 0 (0/166) | 4.302 |
| Xscore_chemscore | 100 (166/166) | 0 (0/166) | 0.564 |
| Goldscore | 100 (166/166) | 0 (0/166) | 8.809 |
| Xscore_goldscore | 100 (166/166) | 0 (0/166) | 0.552 |
| Glidescore | 99.4 (165/166) | 0.60(1/166) | 1.393 |
| **Test set (AstraZeneca-unpublished)** | | | |
| Chemscore | 100 (638/638) | 0 (0/638) | 4.302 |
| Xscore_chemscore | 99.7 (636/638) | 0.30 (0/638) | 0.564 |
| Goldscore | 99.8 (637/166) | 0.20 (1/638) | 8.809 |
| Xscore_goldscore | 99.8 (637/638) | 0.20(1/638) | 0.552 |
| Glidescore | 100 (638/638) | 0 (0/638) | 1.393 |

**Table S2.** Probability of prediction for different scoring function.

| **ChemScore scoring function** | | | | | | | | | | |
| --- | --- | --- | --- | --- | --- | --- | --- | --- | --- | --- |
| **Bin** | **Probability of Inhibitor** | | | | **Probability of non-inhibitor** | | | **p-value** | | |
|  | **Training Set** | | **Test set (Pedersen et al.)** | **Test set (AstraZeneca-unpublished)** | **Training Set** | **Test set (Pedersen et al.)** | **Test set (AstraZeneca-unpublished)** | **Training Set** | **Test set (Pedersen et al.)** | **Test set (AstraZeneca-unpublished)** |
| 0-5 | 0 | | 0 | 0 | 1 | 0 | 1 | 1.00E+000 | NA | 1.00E+000 |
| 5-10 | 0 | | 0 | 0 | 1 | 0 | 1 | 6.68E-001 | NA | 6.87E-001 |
| 10-15 | 0 | | 0 | 0 | 1 | 1 | 1 | 2.51E-002 | 5.21E-001 | 5.68E-002 |
| 15-20 | 0 | | 0 | 0.16 | 1 | 1 | 0.84 | 2.78E-07 | 5.38E-002 | 2.08E-003 |
| 20-25 | 0.10 | | 0.09 | 0.13 | 0.90 | 0.91 | 0.87 | 5.09E-06 | 2.64E-002 | 2.12E-010 |
| 25-30 | 0.29 | | 0.14 | 0.32 | 0.71 | 0.86 | 0.68 | 8.24E-001 | 2.74E-002 | 6.27E-003 |
| 30-35 | 0.70 | | 0.40 | 0.58 | 0.30 | 0.60 | 0.42 | < 2.20E-016 | 1.84E-002 | 4.79E-009 |
| 35-40 | 0.88 | | 0.75 | 0.78 | 0.12 | 0.25 | 0.22 | 5.93E-08 | 1.52E-005 | 1.82E-008 |
| 40-45 | 0.5 | | 0.75 | 0.94 | 0.5 | 0.25 | 0.06 | 1.00E+00 | 0.09873 | 3.14E-006 |
| 45-50 | 1 | | 0 | 1 | 0 | 0 | 0 | 0.6178 | NA | 0.8193 |
| 50-55 | 0 | | 0 | 0 | 0 | 0 | 0 | NA | NA | NA |
| 55-60 | 0 | | 0 | 0 | 0 | 0 | 0 | NA | NA | NA |
| **GoldScore scoring function** | | | | | | | | | | |
| **Bin** | **Probability of Inhibitor** | | | | **Probability of non-inhibitor** | | | **p-value** | | |
|  | **Training Set** | | **Test set (Pedersen et al.)** | **Test set (AstraZeneca-unpublished)** | **Training Set** | **Test set (Pedersen et al.)** | **Test set (AstraZeneca-unpublished)** | **Training Set** | **Test set (Pedersen et al.)** | **Test set (AstraZeneca-unpublished)** |
| 0-5 | 0 | | 0 | 0 | 0 | 0 | 0 | NA | NA | NA |
| 5-10 | 0 | | 0 | 0 | 0 | 0 | 0 | NA | NA | NA |
| 10-15 | 0 | | 0 | 0 | 0 | 0 | 0 | NA | NA | NA |
| 15-20 | 0 | | 0 | 1 | 0 | 0 | 0 | NA | NA | 0.8193 |
| 20-25 | 0 | | 0 | 0 | 1 | 1 | 0 | 1.00E+000 | 1.00E+000 | NA |
| 25-30 | 0 | | 0 | 0 | 1 | 1 | 1 | 1.71E-001 | 9.61E-001 | 4.29E-001 |
| 30-35 | 0 | | 0 | 0 | 1 | 1 | 1 | 6.15E-003 | 9.61E-001 | 1.23E-001 |
| 35-40 | 0 | | 0 | 0.03 | 1 | 1 | 0.97 | 8.65E-06 | 4.22E-002 | 3.27E-005 |
| 40-45 | 0.11 | | 0 | 0.02 | 0.89 | 1 | 0.98 | 7.12E-003 | 2.58E-002 | 5.95E-008 |
| 45-50 | 0.11 | | 0.23 | 0.07 | 0.89 | 0.77 | 0.93 | 5.73E-004 | 2.32E-002 | 4.49E-013 |
| 50-55 | 0.36 | | 0.06 | 0.35 | 0.64 | 0.94 | 0.65 | 1.40E-001 | 1.02E-001 | 4.35E-001 |
| 55-60 | 0.4 | | 0.21 | 0.47 | 0.6 | 0.79 | 0.53 | 5.65E-002 | 6.67E-001 | 4.39E-002 |
| 60-65 | 0.71 | | 0.5 | 0.68 | 0.29 | 0.5 | 0.32 | 1.73E-08 | 1.55E-002 | 1.30E-009 |
| 65-70 | 0.72 | | 0.4 | 0.64 | 0.28 | 0.6 | 0.36 | 2.00E-08 | 3.50E-001 | 1.29E-005 |
| 70-75 | 0.44 | | 0.42 | 0.62 | 0.56 | 0.58 | 0.38 | 4.48E-001 | 3.70E-001 | 8.45E-003 |
| 75-80 | 0.6 | | 1 | 0.75 | 0.4 | 0 | 0.25 | 2.62E-001 | 1.09E-003 | 2.19E-002 |
| 80-85 | 0.33 | | 1 | 0.86 | 0.67 | 0 | 0.14 | 1.00E+000 | 1.18E-001 | 3.03E-002 |
| 85-90 | 0 | | 0.5 | 0.67 | 1 | 0.5 | 0.33 | 1.00E+000 | 6.14E-001 | 6.92E-001 |
| **Xscore(Chemscore) scoring function** | | | | | | | | | | |
| **Bin** | **Probability of Inhibitor** | | | | **Probability of non-inhibitor** | | | **p-value** | | |
|  | **Training Set** | | **Test set (Pedersen et al.)** | **Test set (AstraZeneca-unpublished)** | **Training Set** | **Test set (Pedersen et al.)** | **Test set (AstraZeneca-unpublished)** | **Training Set** | **Test set (Pedersen et al.)** | **Test set (AstraZeneca-unpublished)** |
| 0-0.5 | 0 | | 0 | 0 | 0 | 0 | 0 | NA | NA | NA |
| 0.5-1 | 0 | | 0 | 0 | 0 | 0 | 0 | NA | NA | NA |
| 1-1.5 | 0 | | 0 | 0 | 0 | 0 | 0 | NA | NA | NA |
| 1.5-2 | 0 | | 0 | 0 | 0 | 0 | 0 | NA | NA | NA |
| 2-2.5 | 0 | | 0 | 0 | 0 | 0 | 0 | NA | NA | NA |
| 2.5-3 | 0 | | 0 | 0 | 0 | 0 | 0 | NA | NA | NA |
| 3-3.5 | 0 | | 0 | 0 | 0 | 0 | 0 | NA | NA | NA |
| 3.5-4 | 0 | | 0 | 0 | 1 | 1 | 1 | 1.71E-001 | 1.00E+000 | 1.00E+000 |
| 4-4.5 | 0 | | 0 | 0 | 1 | 1 | 1 | 1.18E-003 | 1.83E-001 | 3.39E-004 |
| 4.5-5 | 0.03 | | 0 | 0.02 | 0.97 | 1 | 0.98 | 1.24E-06 | 2.36E-001 | 1.85E-007 |
| 5-5.5 | 0.05 | | 0.04 | 0.08 | 0.95 | 0.96 | 0.92 | 1.23E-07 | 1.93E-002 | 4.31E-014 |
| 5.5-6 | 0.19 | | 0.16 | 0.25 | 0.81 | 0.84 | 0.75 | 2.97E-002 | 2.20E-001 | 1.49E-004 |
| 6-6.5 | 0.71 | | 0.22 | 0.59 | 0.29 | 0.78 | 0.41 | < 2.20E-016 | 5.72E-001 | 8.29E-010 |
| 6.5-7 | 0.91 | | 0.63 | 0.74 | 0.08 | 0.37 | 0.26 | 1.22E-11 | 3.57E-004 | 3.50E-011 |
| 7-7.5 | 0.83 | | 0.64 | 0.79 | 0.17 | 0.36 | 0.21 | 5.25E-05 | 1.13E-002 | 1.56E-006 |
| 7.5-8 | 1 | | 0.89 | 0.5 | 0 | 0.11 | 0.5 | 6.18E-001 | 1.42E-004 | 8.88E-001 |
| 8-8.5 | 0.8 | | 0 | 0.83 | 0.2 | 1 | 0.17 | 3.34E-002 | 1.00E+000 | 6.81E-002 |
| 8.5-9 | 1 | | 1 | 1 | 0 | 0 | 0 | 6.18E-001 | 5.93E-001 | 4.53E-002 |
| **Xscore(Goldscore) scoring function** | | | | | | | | | | |
| **Bin** | **Probability of Inhibitor** | | | | **Probability of non-inhibitor** | | | **p-value** | | |
|  | **Training Set** | | **Test set (Pedersen et al.)** | **Test set (AstraZeneca-unpublished)** | **Training Set** | **Test set (Pedersen et al.)** | **Test set (AstraZeneca-unpublished)** | **Training Set** | **Test set (Pedersen et al.)** | **Test set (AstraZeneca-unpublished)** |
| 0-0.5 | 0 | | 0 | 0 | 0 | 0 | 0 | NA | NA | NA |
| 0.5-1 | 0 | | 0 | 0 | 0 | 0 | 0 | NA | NA | NA |
| 1-1.5 | 0 | | 0 | 0 | 0 | 0 | 0 | NA | NA | NA |
| 1.5-2 | 0 | | 0 | 0 | 0 | 0 | 0 | NA | NA | NA |
| 2-2.5 | 0 | | 0 | 0 | 0 | 0 | 0 | NA | NA | NA |
| 2.5-3 | 0 | | 0 | 0 | 0 | 0 | 0 | NA | NA | NA |
| 3-3.5 | 0 | | 0 | 0 | 0 | 0 | 0 | NA | NA | NA |
| 3.5-4 | 0 | | 0 | 0 | 1 | 0 | 0 | 0.9363 | NA | NA |
| 4-4.5 | 0 | | 0 | 0 | 1 | 1 | 1 | 5.79E-004 | 3.96E-001 | 2.36E-004 |
| 4.5-5 | 0.01 | | 0 | 0 | 0.99 | 1 | 1 | 1.55E-08 | 5.69E-003 | 4.72E-010 |
| 5-5.5 | 0.04 | | 0.05 | 0.03 | 0.96 | 0.95 | 0.97 | 5.04E-08 | 3.15E-002 | 7.31E-016 |
| 5.5-6 | 0.22 | | 0.09 | 0.32 | 0.77 | 0.91 | 0.68 | 2.25E-001 | 2.08E-002 | 5.30E-002 |
| 6-6.5 | 0.76 | | 0.34 | 0.56 | 0.24 | 0.66 | 0.44 | < 2.20E-016 | 2.35E-001 | 2.50E-007 |
| 6.5-7 | 0.88 | | 0.65 | 0.72 | 0.12 | 0.35 | 0.28 | 4.23E-12 | 1.01E-004 | 3.91E-010 |
| 7-7.5 | 0.92 | | 0.58 | 0.81 | 0.08 | 0.42 | 0.19 | 6.50E-07 | 2.42E-002 | 2.57E-006 |
| 7.5-8 | 0.75 | | 0.8 | 0.93 | 0.25 | 0.2 | 0.07 | 1.18E-001 | 2.53E-002 | 9.12E-005 |
| 8-8.5 | 1 | | 0 | 0.86 | 0 | 1 | 0.14 | 1.34E-001 | 1.00E+000 | 3.03E-002 |
| 8.5-9 | 0 | | 0 | 1 | 0 | 0 | 0 | NA | NA | 2.94E-001 |
| **GlideXP scoring function** | | | | | | | | | | |
| **Bin** | | **Probability of Inhibitor** | | | **Probability of non-inhibitor** | | | **p-value** | | |
|  | | **Training Set** | **Test set (Pedersen et al.)** | **Test set (AstraZeneca-unpublished)** | **Training Set** | **Test set (Pedersen et al.)** | **Test set (AstraZeneca-unpublished)** | **Training Set** | **Test set (Pedersen et al.)** | **Test set (AstraZeneca-unpublished)** |
| -16 - -15.5 | | 0 | 0 | 0 | 0 | 0 | 0 | NA | NA | NA |
| -15.5 - -15 | | 0 | 0 | 0 | 0 | 0 | 0 | NA | NA | NA |
| -15 - -14.5 | | 0 | 0 | 0 | 1 | 0 | 0 | 1.00E+000 | NA | NA |
| -14.5 - -14 | | 0 | 1 | 0 | 0 | 0 | 0 | NA | 5.93E-001 | NA |
| -14 - -13.5 | | 0 | 0 | 0 | 0 | 0 | 0 | NA | NA | NA |
| -13.5 - -13 | | 0 | 0 | 0 | 1 | 0 | 0 | 1.00E+000 | NA | NA |
| -13 - -12.5 | | 0 | 0 | 1 | 0 | 0 | 0 | NA | NA | 8.19E-001 |
| -12.5 - -12 | | 1 | 0 | 1 | 0 | 1 | 0 | 6.12E-001 | 9.61E-001 | 8.19E-001 |
| -12 - -11.5 | | 0 | 0 | 0 | 0 | 1 | 1 | NA | 9.61E-001 | 1.00E+000 |
| -11.5 - -11 | | 0.33 | 1 | 0 | 0.67 | 0 | 1 | 1.00E+000 | 2.30E-004 | 1.00E+000 |
| -11 - -10.5 | | 1 | 0.25 | 0.33 | 0 | 0.75 | 0.67 | 6.12E-001 | 1.00E+000 | 1.00E+000 |
| -10.5 - -10 | | 0 | 0.33 | 0.2 | 1 | 0.67 | 0.8 | 3.80E-001 | 1.00E+000 | 6.83E-001 |
| -10 - -9.5 | | 0.54 | 0.6 | 0.58 | 0.46 | 0.4 | 0.42 | 6.31E-002 | 2.27E-001 | 1.37E-001 |
| -9.5 - -9 | | 0.71 | 0.47 | 0.69 | 0.29 | 0.53 | 0.31 | 2.72E-002 | 1.22E-001 | 4.17E-004 |
| -9 - -8.5 | | 0.68 | 0.27 | 0.55 | 0.32 | 0.73 | 0.45 | 3.10E-05 | 1.00E+000 | 9.93E-002 |
| -8.5 - -8 | | 0.56 | 0.33 | 0.48 | 0.44 | 0.67 | 0.52 | 1.51E-003 | 9.29E-001 | 1.88E-001 |
| -8 - -7.5 | | 0.55 | 0.43 | 0.58 | 0.45 | 0.57 | 0.42 | 1.09E-003 | 2.58E-001 | 1.88E-001 |
| -7.5 - -7 | | 0.41 | 0.24 | 0.6 | 0.59 | 0.76 | 0.4 | 6.88E-002 | 9.97E-001 | 5.75E-005 |
| -7 - -6.5 | | 0.33 | 0.18 | 0.41 | 0.67 | 0.82 | 0.59 | 6.21E-001 | 5.60E-001 | 7.79E-001 |
| -6.5 - -6 | | 0.20 | 0.06 | 0.33 | 0.80 | 0.94 | 0.67 | 3.67E-001 | 8.12E-002 | 3.44E-001 |
| -6 - -5.5 | | 0.12 | 0.17 | 0.14 | 0.88 | 0.83 | 0.86 | 5.33E-002 | 6.44E-001 | 5.20E-005 |
| -5.5 - -5 | | 0.09 | 0 | 0.2 | 0.91 | 1 | 0.8 | 5.57E-003 | 1.43E-001 | 6.93E-003 |
| -5 - -4.5 | | 0.11 | 0.14 | 0.19 | 0.89 | 0.86 | 0.81 | 4.39E-002 | 7.56E-001 | 2.23E-002 |
| -4.5 - -4 | | 0.11 | 0.17 | 0.15 | 0.89 | 0.83 | 0.85 | 6.73E-002 | 9.32E-001 | 4.64E-002 |
| -4 - -3.5 | | 0.11 | 0 | 0.11 | 0.89 | 1 | 0.89 | 1.54E-001 | 1.00E+000 | 2.74E-002 |
| -3.5 - -3 | | 0 | 0 | 0.13 | 1 | 1 | 0.87 | 1.76E-001 | 1.00E+000 | 2.40E-001 |
| -3 - -2.5 | | 0 | 0 | 0 | 1 | 1 | 1 | 6.72E-002 | 6.97E-001 | 5.68E-002 |
| -2.5 - -2 | | 0 | 0 | 0 | 1 | 0 | 1 | 9.40E-001 | NA | 4.29E-001 |
| -2 - -1.5 | | 0 | 0.5 | 0 | 1 | 0.5 | 1 | 1.00E+000 | 1.00E+000 | 1.00E+000 |
| -1.5 - -1 | | 0 | 0 | 0 | 1 | 0 | 0 | 5.02E-001 | NA | NA |
| -1 - -0.5 | | 0 | 0 | 0.5 | 0 | 1 | 0.5 | NA | 1.00E+000 | 1.00E+000 |
| -0.5 - 0 | | 0 | 0 | 0 | 1 | 0 | 0 | 1.00E+000 | NA | NA |
| 0 - 0.5 | | 0 | 0 | 0 | 0 | 0 | 0 | NA | NA | NA |

The scoring function in brackets was used to generate the docking poses.

**Table S3 (a).** Summary of models obtained using different scoring functions for the training set

| **Scoring Function** | **Intersection Point** | **TP** | **TN** | **FP** | **FN** | **Sensitivity** | **Specificity** | **Accuracy** | **G-mean** | **MCC** |
| --- | --- | --- | --- | --- | --- | --- | --- | --- | --- | --- |
| ChemScore | 29.50 | 68 | 261 | 34 | 45 | 0.60 | 0.88 | 0.81 | 0.73 | 0.50 |
| GoldScore | 53.50 | 83 | 221 | 74 | 30 | 0.74 | 0.75 | 0.75 | 0.74 | 0.45 |
| Maestro_docking score(XP) | -6.80 | 89 | 192 | 105 | 23 | 0.80 | 0.65 | 0.69 | 0.72 | 0.39 |
| Consensus |  | 88 | 236 | 59 | 25 | 0.78 | 0.80 | 0.79 | 0.79 | 0.54 |
| Xscore (ChemScore) | 6.15 | 80 | 279 | 16 | 33 | 0.71 | 0.95 | 0.88 | 0.82 | 0.69 |
| Xscore (GoldScore) | 6.10 | 77 | 280 | 15 | 36 | 0.68 | 0.95 | 0.86 | 0.80 | 0.68 |
| **ChemScore+Molecular Weight (MW)+logP (Normalized)** | | | | | | | | | | |
| ChemScore+MW | 0.80 | 91 | 266 | 29 | 22 | 0.81 | 0.90 | 0.88 | 0.85 | 0.69 |
| ChemScore+logP | 1.10 | 77 | 269 | 26 | 36 | 0.68 | 0.91 | 0.85 | 0.79 | 0.61 |
| ChemScore+MW+logP | 1.20 | 85 | 270 | 25 | 28 | 0.75 | 0.92 | 0.87 | 0.83 | 0.67 |
| **Xscore(ChemScore)+Molecular Weight (MW)+logP (Normalized)** | | | | | | | | | | |
| Xscore(ChemScore) +MW | 0.70 | 96 | 263 | 32 | 17 | 0.85 | 0.89 | 0.88 | 0.87 | 0.72 |
| Xscore(ChemScore) +logP | 1.2 | 78 | 282 | 13 | 35 | 0.69 | 0.96 | 0.89 | 0.81 | 0.86 |
| Xscore(ChemScore) +MW+logP | 0.90 | 92 | 266 | 29 | 21 | 0.81 | 0.90 | 0.88 | 0.86 | 0.70 |
| **GoldScore+Molecular Weight (MW)+logP(Normalized)** | | | | | | | | | | |
| GoldScore+MW | 0.40 | 91 | 230 | 65 | 22 | 0.81 | 0.78 | 0.79 | 0.79 | 0.58 |
| GoldScore+logP | 0.70 | 91 | 259 | 36 | 22 | 0.81 | 0.88 | 0.86 | 0.84 | 0.66 |
| GoldScore+MW+logP | 0.70 | 99 | 250 | 45 | 14 | 0.88 | 0.85 | 0.86 | 0.86 | 0.68 |
| **Xscore(GoldScore)+Molecular Weight (MW)+logP(Normalized)** | | | | | | | | | | |
| Xscore(GoldScore) +MW | 0.5 | 97 | 251 | 44 | 16 | 0.86 | 0.85 | 0.85 | 0.86 | 0.67 |
| Xscore(GoldScore)+logP | ---- |  |  |  |  |  |  |  |  |  |
| Xscore(GoldScore)+MW+logP | 0.80 | 97 | 267 | 28 | 16 | 0.86 | 0.91 | 0.89 | 0.88 | 0.74 |

The scoring function in brackets was used to generate the docking poses.

**Table S3 (b).** Summary of models obtained using different scoring functions for the test set (Pedersen et al.)

| **Scoring Function** | **Intersection Point** | **TP** | **TN** | **FP** | **FN** | **Sensitivity** | **Specificity** | **Accuracy** | **G-mean** | **MCC** |
| --- | --- | --- | --- | --- | --- | --- | --- | --- | --- | --- |
| ChemScore | 29.50 | 35 | 86 | 36 | 9 | 0.80 | 0.71 | 0.73 | 0.75 | 0.45 |
| GoldScore | 53.50 | 37 | 69 | 53 | 7 | 0.84 | 0.57 | 0.64 | 0.69 | 0.36 |
| Maestro_docking score(XP) | -6.80 | 38 | 49 | 73 | 6 | 0.86 | 0.40 | 0.52 | 0.59 | 0.25 |
| Consensus |  | 38 | 67 | 55 | 6 | 0.86 | 0.55 | 0.63 | 0.69 | 0.37 |
| **Xscore** | | | | | | | | | | |
| Xscore (ChemScore) | 6.15 | 38 | 82 | 40 | 6 | 0.86 | 0.67 | 0.72 | 0.76 | 0.47 |
| Xscore (GoldScore) | 6.10 | 35 | 83 | 39 | 9 | 0.80 | 0.68 | 0.71 | 0.74 | 0.42 |
| **ChemScore+Molecular Weight (MW)+ logP(Normalized)** | | | | | | | | | | |
| ChemScore+MW | 0.80 | 34 | 109 | 13 | 10 | 0.77 | 0.89 | 0.86 | 0.83 | 0.65 |
| ChemScore+logP | 1.10 | 31 | 122 | 0 | 13 | 0.71 | 1.00 | 0.92 | 0.84 | 0.80 |
| ChemScore+MW+logP | 1.20 | 36 | 120 | 2 | 8 | 0.82 | 0.98 | 0.94 | 0.90 | 0.84 |
| **Xscore(ChemScore)+Molecular Weight (MW)+ logP(Normalized)** | | | | | | | | | | |
| Xscore(ChemScore)+MW | 0.70 | 38 | 100 | 22 | 6 | 0.86 | 0.82 | 0.83 | 0.84 | 0.63 |
| Xscore(ChemScore)+logP | 1.2 | 24 | 113 | 9 | 20 | 0.55 | 0.93 | 0.83 | 0.71 | 0.52 |
| Xscore(ChemScore)+MW+logP | 0.90 | 37 | 106 | 16 | 7 | 0.84 | 0.87 | 0.86 | 0.86 | 0.67 |
| **GoldScore+MolecularWeight (MW)+ logP(Normalized)** | | | | | | | | | | |
| GoldScore+MW | 0.40 | 35 | 94 | 28 | 9 | 0.80 | 0.77 | 0.78 | 0.78 | 0.52 |
| Goldsscore+logP | 0.70 | 34 | 106 | 16 | 10 | 0.77 | 0.87 | 0.84 | 0.82 | 0.62 |
| GoldScore+MW+logP | 0.70 | 35 | 110 | 12 | 9 | 0.80 | 0.90 | 0.87 | 0.85 | 0.68 |
| **Xscore(GoldScore)+Molecular Weight (MW)+ logP(Normalized)** | | | | | | | | | | |
| Xscore(GoldScore)+MW | 0.50 | 38 | 95 | 27 | 6 | 0.86 | 0.78 | 0.80 | 0.82 | 0.58 |
| Xscore(GoldScore)+logP | ---- |  |  |  |  |  |  |  |  |  |
| Xscore(GoldScore)+MW+logP | 0.80 | 40 | 104 | 18 | 4 | 0.91 | 0.85 | 0.87 | 0.88 | 0.71 |

The scoring function in brackets was used to generate the docking poses.

**Table S3 (c).** Summary of models obtained using different scoring function for the the test set (AstraZeneca-unpublished)

| **Scoring Function** | **Intersection Point** | **TP** | **TN** | **FP** | **FN** | **Sensitivity** | **Specificity** | **Accuracy** | **G-mean** | **MCC** |
| --- | --- | --- | --- | --- | --- | --- | --- | --- | --- | --- |
| ChemScore | 29.50 | 162 | 297 | 93 | 86 | 0.65 | 0.76 | 0.72 | 0.71 | 0.41 |
| GoldScore | 53.50 | 213 | 240 | 150 | 35 | 0.86 | 0.62 | 0.71 | 0.73 | 0.47 |
| Maestro_docking score(XP) | -6.80 | 199 | 205 | 185 | 49 | 0.80 | 0.53 | 0.63 | 0.65 | 0.33 |
| Consensus |  | 211 | 257 | 133 | 37 | 0.85 | 0.66 | 0.73 | 0.75 | 0.50 |
| **Xscore** | | | | | | | | | | |
| Xscore (ChemScore) | 6.15 | 179 | 310 | 80 | 69 | 0.72 | 0.80 | 0.77 | 0.76 | 0.51 |
| Xscore (GoldScore) | 6.10 | 175 | 306 | 84 | 73 | 0.71 | 0.79 | 0.75 | 0.74 | 0.49 |
| **ChemScore+Molecular Weight (MW)+ logP(Normalized)** | | | | | | | | | | |
| ChemScore+MW | 0.80 | 121 | 347 | 43 | 127 | 0.49 | 0.89 | 0.73 | 0.66 | 0.42 |
| ChemScore+logP | 1.10 | 121 | 352 | 38 | 127 | 0.49 | 0.90 | 0.74 | 0.66 | 0.44 |
| ChemScore+MW+logP | 1.20 | 131 | 356 | 34 | 117 | 0.53 | 0.91 | 0.76 | 0.69 | 0.49 |
| **Xscore(ChemScore)+Molecular Weight (MW)+ logP(Normalized)** | | | | | | | | | | |
| Xscore(ChemScore)+MW | 0.70 | 134 | 344 | 46 | 114 | 0.54 | 0.88 | 0.75 | 0.69 | 0.46 |
| Xscore(ChemScore)+logP | 1.2 | 104 | 363 | 27 | 144 | 0.42 | 0.93 | 0.73 | 0.63 | 0.42 |
| Xscore(ChemScore)+MW+logP | 0.90 | 143 | 346 | 44 | 105 | 0.58 | 0.89 | 0.77 | 0.72 | 0.50 |
| **GoldScore+Molecular Weight (MW)+ logP(Normalized)** | | | | | | | | | | |
| GoldScore+MW | 0.40 | 165 | 308 | 82 | 83 | 0.67 | 0.79 | 0.74 | 0.73 | 0.46 |
| Goldsscore+logP | 0.70 | 142 | 342 | 48 | 106 | 0.57 | 0.88 | 0.76 | 0.71 | 0.48 |
| GoldScore+MW+logP | 0.70 | 153 | 332 | 58 | 95 | 0.62 | 0.85 | 0.76 | 0.73 | 0.49 |
| **Xscore(GoldScore)+Molecular Weight (MW)+ logP(Normalized)** | | | | | | | | | | |
| Xscore(GoldScore)+MW | 0.50 | 156 | 328 | 62 | 92 | 0.63 | 0.84 | 0.76 | 0.73 | 0.48 |
| Xscore(GoldScore)+logP | ---- |  |  |  |  |  |  |  |  |  |
| Xscore(GoldScore)+MW+logP | 0.80 | 156 | 345 | 45 | 92 | 0.63 | 0.89 | 0.79 | 0.75 | 0.54 |

The scoring function in brackets was used to generate the docking poses.

**Table S4 (a).** Machine learning models obtained using ChemScore scoring function combined with physicochemical properties as descriptors for the training set

| **Descriptor** | **Machine Learning method** | **TP** | **TN** | **FP** | **FN** | **Sensitivity** | **Specificity** | **Accuracy** | **G-mean** | **MCC** |
| --- | --- | --- | --- | --- | --- | --- | --- | --- | --- | --- |
| **MolecularWeight (MW)+logP** | | | | | | | | | | |
| MW+logP | Naive Bayes | 63 | 284 | 11 | 50 | 0.56 | 0.96 | 0.85 | 0.73 | 0.60 |
| MW+logP | LibSVM | 37 | 287 | 8 | 76 | 0.33 | 0.97 | 0.79 | 0.56 | 0.43 |
| MW+logP | J48 | 88 | 265 | 30 | 25 | 0.78 | 0.90 | 0.87 | 0.84 | 0.67 |
| MW+logP | RF | 95 | 266 | 29 | 18 | 0.84 | 0.90 | 0.88 | 0.87 | 0.72 |
| MW+logP | REPTree | 85 | 265 | 30 | 28 | 0.75 | 0.90 | 0.86 | 0.82 | 0.65 |
| **ChemScore+** MolecularWeight **(MW)** | | | | | | | | | | |
| ChemScore+MW | Naive Bayes | 75 | 272 | 23 | 38 | 0.66 | 0.92 | 0.85 | 0.78 | 0.61 |
| ChemScore+MW | LibSVM | 53 | 284 | 11 | 60 | 0.47 | 0.96 | 0.83 | 0.67 | 0.53 |
| ChemScore+MW | J48 | 77 | 280 | 15 | 36 | 0.68 | 0.95 | 0.88 | 0.80 | 0.68 |
| ChemScore+MW | RF | 85 | 267 | 28 | 28 | 0.75 | 0.91 | 0.86 | 0.83 | 0.66 |
| ChemScore+MW | REPTree | 78 | 273 | 22 | 35 | 0.69 | 0.93 | 0.86 | 0.80 | 0.64 |
| **ChemScore+logP** | | | | | | | | | | |
| ChemScore+logP | Naive Bayes | 78 | 263 | 32 | 35 | 0.69 | 0.89 | 0.84 | 0.78 | 0.59 |
| ChemScore+logP | LibSVM | 34 | 291 | 4 | 79 | 0.30 | 0.99 | 0.80 | 0.54 | 0.44 |
| ChemScore+logP | J48 | 69 | 268 | 27 | 44 | 0.61 | 0.91 | 0.83 | 0.74 | 0.55 |
| ChemScore+logP | RF | 75 | 256 | 39 | 38 | 0.66 | 0.87 | 0.81 | 0.76 | 0.53 |
| ChemScore+logP | REPTree | 69 | 267 | 28 | 44 | 0.61 | 0.91 | 0.82 | 0.74 | 0.54 |
| **ChemScore+ Molecular Weight (MW)+logP** | | | | | | | | | | |
| ChemScore+MW+logP | Naive Bayes | 83 | 271 | 24 | 30 | 0.74 | 0.92 | 0.87 | 0.82 | 0.66 |
| ChemScore+MW+logP | LibSVM | 53 | 288 | 7 | 60 | 0.47 | 0.98 | 0.84 | 0.68 | 0.56 |
| ChemScore+MW+logP | J48 | 93 | 273 | 22 | 20 | 0.82 | 0.93 | 0.90 | 0.87 | 0.74 |
| ChemScore+MW+logP | RF | 91 | 267 | 28 | 22 | 0.81 | 0.91 | 0.88 | 0.85 | 0.70 |
| ChemScore+MW+logP | REPTree | 83 | 269 | 26 | 30 | 0.73 | 0.91 | 0.86 | 0.82 | 0.65 |
| **Xscore(ChemScore)** | | | | | | | | | | |
| **Xscore_C+Molecular Weight (MW)** | | | | | | | | | | |
| Xscore_C+MW | Naive Bayes | 82 | 276 | 19 | 31 | 0.73 | 0.94 | 0.88 | 0.82 | 0.69 |
| Xscore_C+MW | LibSVM | 72 | 286 | 9 | 41 | 0.64 | 0.97 | 0.88 | 0.79 | 0.68 |
| Xscore_C+MW | J48 | 85 | 276 | 19 | 28 | 0.75 | 0.94 | 0.88 | 0.84 | 0.71 |
| Xscore_C+MW | RF | 85 | 267 | 28 | 28 | 0.75 | 0.91 | 0.86 | 0.83 | 0.66 |
| Xscore_C+MW | REPTree | 82 | 274 | 21 | 31 | 0.73 | 0.93 | 0.87 | 0.82 | 0.67 |
| **Xscore_C+logP** | | | | | | | | | | |
| Xscore_C+logP | Naive Bayes | 80 | 276 | 19 | 33 | 0.71 | 0.94 | 0.87 | 0.81 | 0.67 |
| Xscore_C+logP | LibSVM | 67 | 285 | 10 | 46 | 0.59 | 0.97 | 0.86 | 0.76 | 0.64 |
| Xscore_C+logP | J48 | 83 | 270 | 25 | 30 | 0.73 | 0.92 | 0.87 | 0.82 | 0.66 |
| Xscore_C+logP | RF | 79 | 273 | 22 | 34 | 0.70 | 0.93 | 0.86 | 0.80 | 0.65 |
| Xscore_C+logP | REPTree | 79 | 273 | 22 | 34 | 0.70 | 0.93 | 0.86 | 0.80 | 0.65 |
| **Xscore_C+MW+logP** | | | | | | | | | | |
| Xscore_C+MW+logP | Naive Bayes | 80 | 282 | 13 | 30 | 0.73 | 0.96 | 0.89 | 0.83 | 0.72 |
| Xscore_C+MW+logP | LibSVM | 68 | 288 | 7 | 45 | 0.60 | 0.98 | 0.87 | 0.77 | 0.67 |
| Xscore_C+MW+logP | J48 | 88 | 272 | 23 | 25 | 0.78 | 0.92 | 0.88 | 0.85 | 0.70 |
| Xscore_C+MW+logP | RF | 90 | 271 | 24 | 23 | 0.80 | 0.92 | 0.88 | 0.86 | 0.71 |
| Xscore_C+MW+logP | REPTree | 89 | 269 | 26 | 24 | 0.79 | 0.91 | 0.88 | 0.85 | 0.70 |

The scoring function in brackets was used to generate the docking poses. Xscore_C means Xscore(ChemScore).

**Table S4 (b).** Machine learning models obtained using ChemScore scoring function combined with physicochemical properties as descriptors for the test set (Pedersen et al.)

| **Descriptor** | **Machine Learning method** | **TP** | **TN** | **FP** | **FN** | **Sensitivity** | **Specificity** | **Accuracy** | **G-mean** | **MCC** |
| --- | --- | --- | --- | --- | --- | --- | --- | --- | --- | --- |
| **MolecularWeight (MW)+logP** | | | | | | | | | | |
| MW+logP | Naive Bayes | 31 | 110 | 12 | 13 | 0.70 | 0.90 | 0.85 | 0.80 | 0.61 |
| MW+logP | LibSVM | 26 | 112 | 10 | 18 | 0.59 | 0.92 | 0.83 | 0.74 | 0.55 |
| MW+logP | J48 | 33 | 107 | 15 | 11 | 0.75 | 0.88 | 0.84 | 0.81 | 0.61 |
| MW+logP | RF | 35 | 104 | 18 | 9 | 0.80 | 0.85 | 0.84 | 0.82 | 0.61 |
| MW+logP | REPTree | 39 | 101 | 21 | 5 | 0.89 | 0.83 | 0.84 | 0.86 | 0.66 |
| **ChemScore+ MolecularWeight (MW)** | | | | | | | | | | |
| ChemScore+MW | Naive Bayes | 33 | 103 | 19 | 11 | 0.75 | 0.84 | 0.82 | 0.80 | 0.57 |
| ChemScore+MW | LibSVM | 29 | 111 | 11 | 15 | 0.66 | 0.91 | 0.84 | 0.77 | 0.59 |
| ChemScore+MW | J48 | 32 | 108 | 14 | 12 | 0.73 | 0.89 | 0.84 | 0.80 | 0.60 |
| ChemScore+MW | RF | 32 | 100 | 22 | 12 | 0.73 | 0.82 | 0.80 | 0.77 | 0.52 |
| ChemScore+MW | REPTree | 32 | 106 | 16 | 12 | 0.73 | 0.87 | 0.83 | 0.79 | 0.58 |
| **ChemScore+logP** | | | | | | | | | | |
| ChemScore+logP | Naive Bayes | 37 | 100 | 22 | 7 | 0.84 | 0.82 | 0.83 | 0.83 | 0.61 |
| ChemScore+logP | LibSVM | 24 | 114 | 8 | 20 | 0.55 | 0.93 | 0.83 | 0.71 | 0.54 |
| ChemScore+logP | J48 | 38 | 93 | 29 | 6 | 0.86 | 0.76 | 0.79 | 0.81 | 0.56 |
| ChemScore+logP | RF | 30 | 96 | 26 | 14 | 0.68 | 0.79 | 0.76 | 0.73 | 0.44 |
| ChemScore+logP | REPTree | 31 | 102 | 20 | 13 | 0.70 | 0.84 | 0.80 | 0.77 | 0.52 |
| **ChemScore+ Molecular Weight (MW) +logP** | | | | | | | | | | |
| ChemScore+MW+logP | Naive Bayes | 35 | 104 | 18 | 9 | 0.80 | 0.85 | 0.84 | 0.82 | 0.61 |
| ChemScore+MW+logP | LibSVM | 29 | 111 | 11 | 15 | 0.66 | 0.91 | 0.84 | 0.77 | 0.59 |
| ChemScore+MW+logP | J48 | 33 | 107 | 15 | 11 | 0.75 | 0.88 | 0.84 | 0.81 | 0.61 |
| ChemScore+MW+logP | RF | 36 | 103 | 19 | 8 | 0.82 | 0.84 | 0.84 | 0.83 | 0.62 |
| ChemScore+MW+logP | REPTree | 40 | 100 | 22 | 4 | 0.91 | 0.82 | 0.84 | 0.86 | 0.66 |
| **Xscore(ChemScore)** | | | | | | | | | | |
| **Xscore_C+ Molecular Weight (MW)** | | | | | | | | | | |
| Xscore_C+MW | Naive Bayes | 37 | 97 | 25 | 7 | 0.84 | 0.80 | 0.81 | 0.82 | 0.58 |
| Xscore_C+MW | LibSVM | 34 | 100 | 22 | 10 | 0.77 | 0.82 | 0.81 | 0.80 | 0.55 |
| Xscore_C+MW | J48 | 37 | 97 | 25 | 7 | 0.84 | 0.80 | 0.81 | 0.82 | 0.58 |
| Xscore_C+MW | RF | 39 | 86 | 36 | 5 | 0.89 | 0.70 | 0.75 | 0.79 | 0.52 |
| Xscore_C+MW | REPTree | 38 | 86 | 36 | 6 | 0.86 | 0.70 | 0.75 | 0.78 | 0.50 |
| Xscore_C+logP | | | | | | | | | | |
| Xscore_C+logP | Naive Bayes | 39 | 99 | 23 | 5 | 0.89 | 0.81 | 0.83 | 0.85 | 0.64 |
| Xscore_C+logP | LibSVM | 34 | 104 | 18 | 10 | 0.77 | 0.85 | 0.83 | 0.81 | 0.59 |
| Xscore_C+logP | J48 | 38 | 80 | 42 | 6 | 0.86 | 0.66 | 0.71 | 0.75 | 0.46 |
| Xscore_C+logP | RF | 35 | 93 | 29 | 9 | 0.80 | 0.76 | 0.77 | 0.78 | 0.51 |
| Xscore_C+logP | REPTree | 38 | 79 | 43 | 6 | 0.86 | 0.65 | 0.70 | 0.75 | 0.45 |
| Xscore_C+ Molecular Weight (MW)+logP | | | | | | | | | | |
| Xscore_C+MW+logP | Naive Bayes | 38 | 99 | 23 | 6 | 0.86 | 0.81 | 0.83 | 0.84 | 0.62 |
| Xscore_C+MW+logP | LibSVM | 36 | 108 | 14 | 8 | 0.82 | 0.89 | 0.87 | 0.85 | 0.68 |
| Xscore_C+MW+logP | J48 | 37 | 93 | 29 | 7 | 0.84 | 0.76 | 0.78 | 0.80 | 0.54 |
| Xscore_C+MW+logP | RF | 34 | 100 | 22 | 10 | 0.77 | 0.82 | 0.81 | 0.80 | 0.55 |
| Xscore_C+MW+logP | REPTree | 36 | 90 | 32 | 8 | 0.82 | 0.74 | 0.76 | 0.78 | 0.50 |

The scoring function in brackets was used to generate the docking poses. Xscore_C means Xscore(ChemScore).

**Table S4 (c).** Machine learning models obtained using ChemScore scoring function combined with physicochemical properties as descriptors for the test set (AstraZeneca-unpublished)

| **Descriptor** | **Machine Learning method** | **TP** | **TN** | **FP** | **FN** | **Sensitivity** | **Specificity** | **Accuracy** | **G-mean** | **MCC** |
| --- | --- | --- | --- | --- | --- | --- | --- | --- | --- | --- |
| **MolecularWeight (MW)+logP** | | | | | | | | | | |
| MW+logP | Naive Bayes | 241 | 136 | 254 | 7 | 0.97 | 0.35 | 0.59 | 0.58 | 0.38 |
| MW+logP | LibSVM | 222 | 266 | 124 | 26 | 0.90 | 0.68 | 0.77 | 0.78 | 0.57 |
| MW+logP | J48 | 136 | 247 | 143 | 112 | 0.55 | 0.63 | 0.60 | 0.59 | 0.18 |
| MW+logP | RF | 152 | 234 | 156 | 96 | 0.61 | 0.60 | 0.61 | 0.61 | 0.21 |
| MW+logP | REPTree | 240 | 204 | 186 | 8 | 0.97 | 0.52 | 0.70 | 0.71 | 0.51 |
| ChemScore+ MolecularWeight (MW) | | | | | | | | | | |
| ChemScore+MW | Naive Bayes | 201 | 302 | 88 | 47 | 0.81 | 0.77 | 0.79 | 0.79 | 0.57 |
| ChemScore+MW | LibSVM | 155 | 332 | 58 | 93 | 0.81 | 0.77 | 0.79 | 0.79 | 0.57 |
| ChemScore+MW | J48 | 176 | 328 | 62 | 72 | 0.71 | 0.84 | 0.79 | 0.77 | 0.56 |
| ChemScore+MW | RF | 190 | 298 | 92 | 58 | 0.77 | 0.76 | 0.77 | 0.77 | 0.52 |
| ChemScore+MW | REPTree | 172 | 333 | 57 | 76 | 0.69 | 0.85 | 0.79 | 0.77 | 0.56 |
| ChemScore+logP | | | | | | | | | | |
| ChemScore+logP | Naive Bayes | 238 | 118 | 272 | 10 | 0.96 | 0.30 | 0.56 | 0.54 | 0.32 |
| ChemScore+logP | LibSVM | 204 | 230 | 160 | 44 | 0.82 | 0.59 | 0.68 | 0.70 | 0.41 |
| ChemScore+logP | J48 | 1818 | 93 | 297 | 67 | 0.96 | 0.24 | 0.84 | 0.48 | 0.30 |
| ChemScore+logP | RF | 170 | 122 | 268 | 78 | 0.69 | 0.31 | 0.46 | 0.46 | 0.00 |
| ChemScore+logP | REPTree | 212 | 214 | 176 | 36 | 0.86 | 0.55 | 0.67 | 0.69 | 0.40 |
| ChemScore+ Molecular Weight (MW)+logP | | | | | | | | | | |
| ChemScore+MW+logP | Naive Bayes | 238 | 144 | 246 | 10 | 0.96 | 0.37 | 0.60 | 0.60 | 0.38 |
| ChemScore+MW+logP | LibSVM | 211 | 284 | 106 | 37 | 0.85 | 0.73 | 0.78 | 0.79 | 0.56 |
| ChemScore+MW+logP | J48 | 136 | 247 | 143 | 112 | 0.55 | 0.63 | 0.60 | 0.59 | 0.18 |
| ChemScore+MW+logP | RF | 204 | 228 | 162 | 44 | 0.82 | 0.59 | 0.68 | 0.69 | 0.40 |
| ChemScore+MW+logP | REPTree | 239 | 212 | 178 | 9 | 0.96 | 0.54 | 0.71 | 0.72 | 0.52 |
| Xscore(ChemScore) | | | | | | | | | | |
| Xscore_C+ Molecular Weight (MW) | | | | | | | | | | |
| Xscore_C+MW | Naive Bayes | 129 | 345 | 45 | 119 | 0.52 | 0.89 | 0.74 | 0.68 | 0.44 |
| Xscore_C+MW | LibSVM | 106 | 358 | 32 | 142 | 0.43 | 0.92 | 0.73 | 0.63 | 0.41 |
| Xscore_C+MW | J48 | 129 | 348 | 42 | 120 | 0.52 | 0.89 | 0.75 | 0.68 | 0.45 |
| Xscore_C+MW | RF | 114 | 327 | 63 | 104 | 0.52 | 0.84 | 0.73 | 0.66 | 0.38 |
| Xscore_C+MW | REPTree | 175 | 325 | 65 | 73 | 0.71 | 0.83 | 0.78 | 0.77 | 0.54 |
| Xscore_C+logP | | | | | | | | | | |
| Xscore_C+logP | Naive Bayes | 223 | 203 | 187 | 25 | 0.90 | 0.52 | 0.67 | 0.68 | 0.43 |
| Xscore_C+logP | LibSVM | 168 | 326 | 64 | 80 | 0.68 | 0.84 | 0.77 | 0.75 | 0.52 |
| Xscore_C+logP | J48 | 141 | 346 | 44 | 107 | 0.57 | 0.89 | 0.76 | 0.71 | 0.49 |
| Xscore_C+logP | RF | 132 | 325 | 65 | 116 | 0.53 | 0.83 | 0.72 | 0.67 | 0.39 |
| Xscore_C+logP | REPTree | 178 | 310 | 80 | 70 | 0.72 | 0.80 | 0.77 | 0.76 | 0.51 |
| Xscore_C+ Molecular Weight (MW)+logP | | | | | | | | | | |
| Xscore_C+MW+logP | Naive Bayes | 226 | 242 | 148 | 22 | 0.91 | 0.62 | 0.73 | 0.75 | 0.53 |
| Xscore_C+MW+logP | LibSVM | 170 | 335 | 55 | 78 | 0.69 | 0.86 | 0.79 | 0.77 | 0.56 |
| Xscore_C+MW+logP | J48 | 167 | 331 | 59 | 81 | 0.67 | 0.85 | 0.78 | 0.76 | 0.53 |
| Xscore_C+MW+logP | RF | 183 | 292 | 98 | 65 | 0.74 | 0.75 | 0.75 | 0.74 | 0.48 |
| Xscore_C+MW+logP | REPTree | 175 | 325 | 65 | 73 | 0.71 | 0.83 | 0.78 | 0.77 | 0.54 |

The scoring function in brackets was used to generate the docking poses. Xscore_C means Xscore(ChemScore).

**Table S5 (a).** Summary of models obtained using PLIF results for the training set

| **Scoring Function** | **Critical value** | **TP** | **TN** | **FP** | **FN** | **Sensitivity** | **Specificity** | **Accuracy** | **G-mean** | **MCC** | **Precision** |
| --- | --- | --- | --- | --- | --- | --- | --- | --- | --- | --- | --- |
| Residues | 0.105 | 86 | 220 | 75 | 27 | 0.76 | 0.75 | 0.75 | 0.75 | 0.46 | 0.53 |
| Resudues+Interaction Type | 0.0739 | 83 | 219 | 76 | 30 | 0.734 | 0.74 | 0.74 | 0.74 | 0.44 | 0.52 |
| Residue+functional groups | 0.0316 | 91 | 235 | 60 | 22 | 0.82 | 0.80 | 0.80 | 0.80 | 0.56 | 0.60 |
| **Sequential approach—TP+FP--ChemScore** | | | | | | | | | | | |
| Residues | 0.105 | 57 | 281 | 14 | 56 | 0.50 | 0.95 | 0.83 | 0.69 | 0.54 | 0.80 |
| Resudues+Interaction Type | 0.0739 | 56 | 281 | 14 | 57 | 0.50 | 0.95 | 0.83 | 0.69 | 0.53 | 0.80 |
| Residue+functional groups | 0.0316 | 58 | 286 | 9 | 55 | 0.51 | 0.97 | 0.84 | 0.71 | 0.58 | 0.87 |

**Table S5 (b).** Summary of models obtained using PLIF results for the test set (Pedersen et. al)

| **Scoring Function** | **Critical value** | **TP** | **TN** | **FP** | **FN** | **Sensitivity** | **Specificity** | **Accuracy** | **G-mean** | **MCC** | **Precision** |
| --- | --- | --- | --- | --- | --- | --- | --- | --- | --- | --- | --- |
| Residues | 0.105 | 41 | 80 | 32 | 3 | 0.93 | 0.71 | 0.78 | 0.82 | 0.58 | 0.56 |
| Resudues+Interaction Type | 0.0739 | 37 | 82 | 40 | 7 | 0.84 | 0.67 | 0.72 | 0.75 | 0.45 | 0.48 |
| Residue+functional groups | 0.0316 | 33 | 95 | 27 | 11 | 0.75 | 0.78 | 0.77 | 0.76 | 0.49 | 0.55 |
| **Sequential approach—TP+FP--ChemScore** | | | | | | | | | | | |
| Residues | 0.105 | 33 | 109 | 13 | 11 | 0.75 | 0.89 | 0.86 | 0.82 | 0.64 | 0.72 |
| Resudues+Interaction Type | 0.0739 | 29 | 110 | 12 | 15 | 0.66 | 0.90 | 0.84 | 0.77 | 0.57 | 0.71 |
| Residue+functional groups | 0.0316 | 28 | 111 | 11 | 16 | 0.64 | 0.91 | 0.84 | 0.76 | 0.57 | 0.72 |

**Table S5 (c).** Summary of models obtained using PLIF results for the test set (AstraZeneca-unpublished)

| **Scoring Function** | **Critical value** | **TP** | **TN** | **FP** | **FN** | **Sensitivity** | **Specificity** | **Accuracy** | **G-mean** | **MCC** | **Precision** | |
| --- | --- | --- | --- | --- | --- | --- | --- | --- | --- | --- | --- | --- |
| Residues | 0.105 | 205 | 287 | 103 | 43 | 0.83 | 0.74 | 0.77 | 0.78 | 0.55 | 0.67 | |
| Resudues+Interaction Type | 0.0739 | 207 | 290 | 100 | 41 | 0.84 | 0.74 | 0.78 | 0.79 | 0.56 | 0.67 | |
| Residue+functional groups | 0.0316 | 191 | 306 | 84 | 57 | 0.77 | 0.79 | 0.78 | 0.78 | 0.55 | 0.70 | |
| **Sequential approach—TP+FP--ChemScore** | | | | | | | | | | | |  |
| Residues | 0.105 | 135 | 350 | 40 | 113 | 0.54 | 0.90 | 0.76 | 0.70 | 0.48 | 0.77 | |
| Resudues+Interaction Type | 0.0739 | 138 | 355 | 35 | 110 | 0.56 | 0.91 | 0.77 | 0.71 | 0.51 | 0.80 | |
| Residue+functional groups | 0.0316 | 131 | 356 | 34 | 117 | 0.53 | 0.91 | 0.76 | 0.69 | 0.49 | 0.79 | |

**Figures**

**Figure S1.** Residues which show hydrophobic interactions with a high interaction rate and a low root mean square fluctuation.


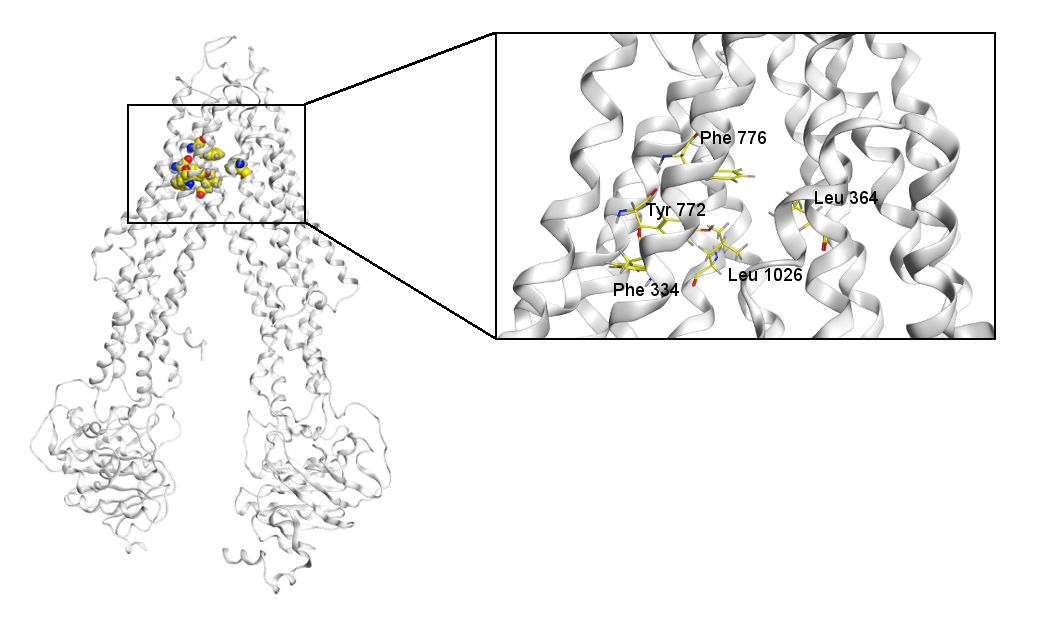


**Figure S2.** CNS representation of the training set compounds based on MACCS Tc similarity threshold of 0.70. Communities with at least five representative members are color coded. Also shown below are the few exemplary compound (with their IC50 value in µM) of highlighted communities.


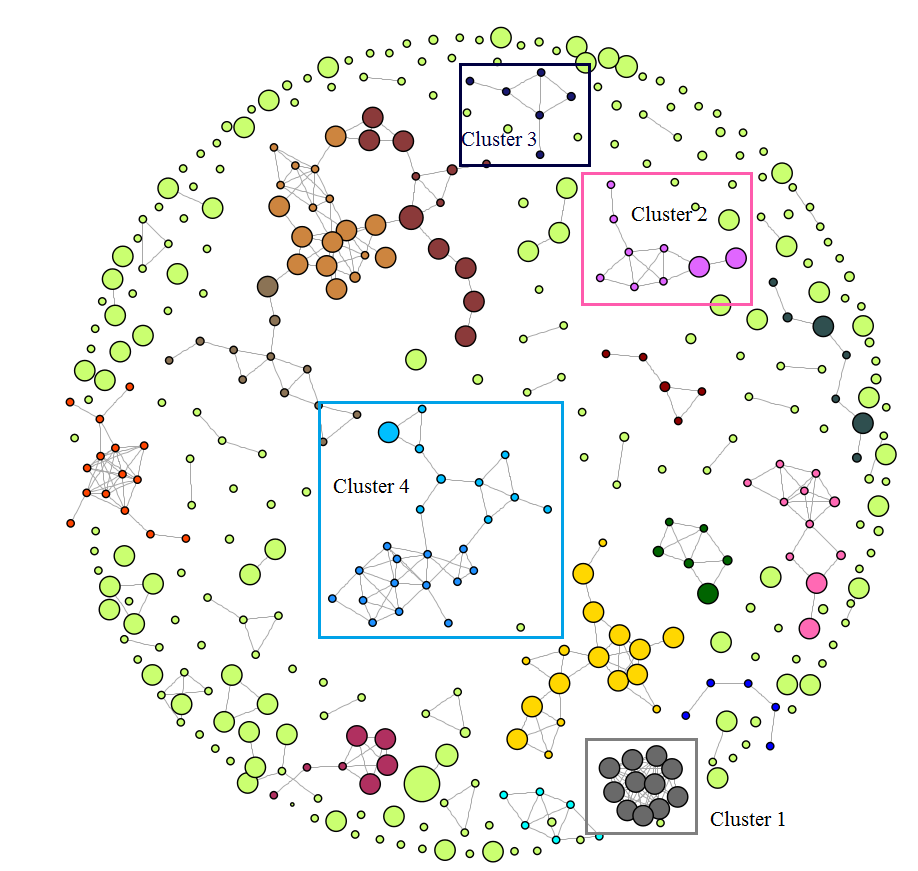

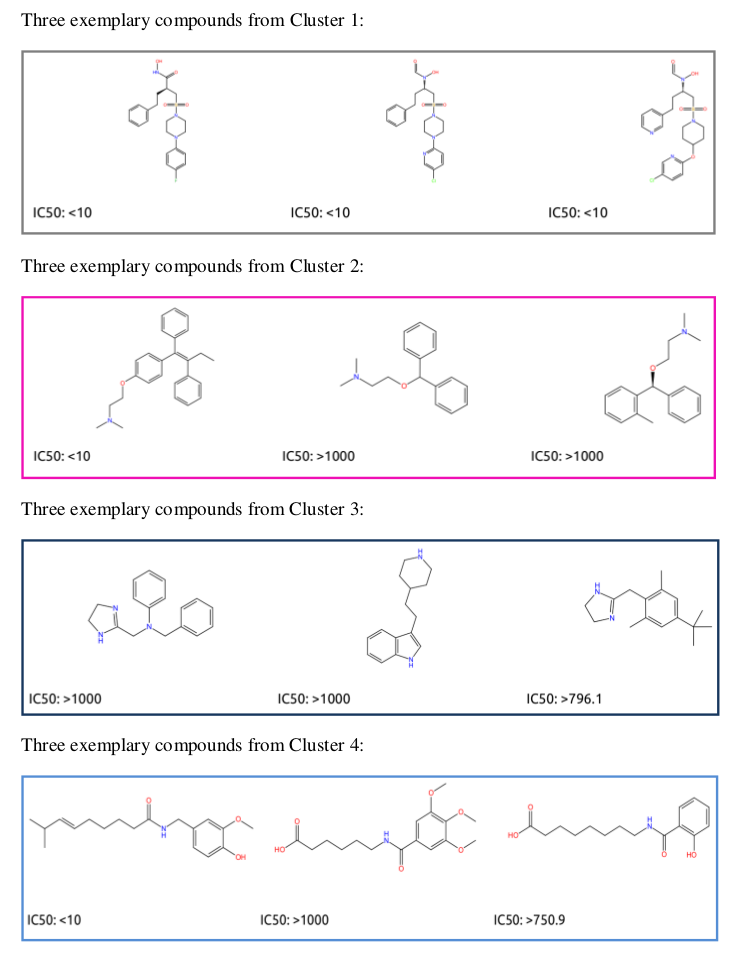


**Figure S3.** CNS representation of the test set compounds (Pedersen et al.) based on MACCS Tc similarity threshold of 0.70. Communities with at least five representative members are color coded. Also shown are the few exemplary compound of these communities.


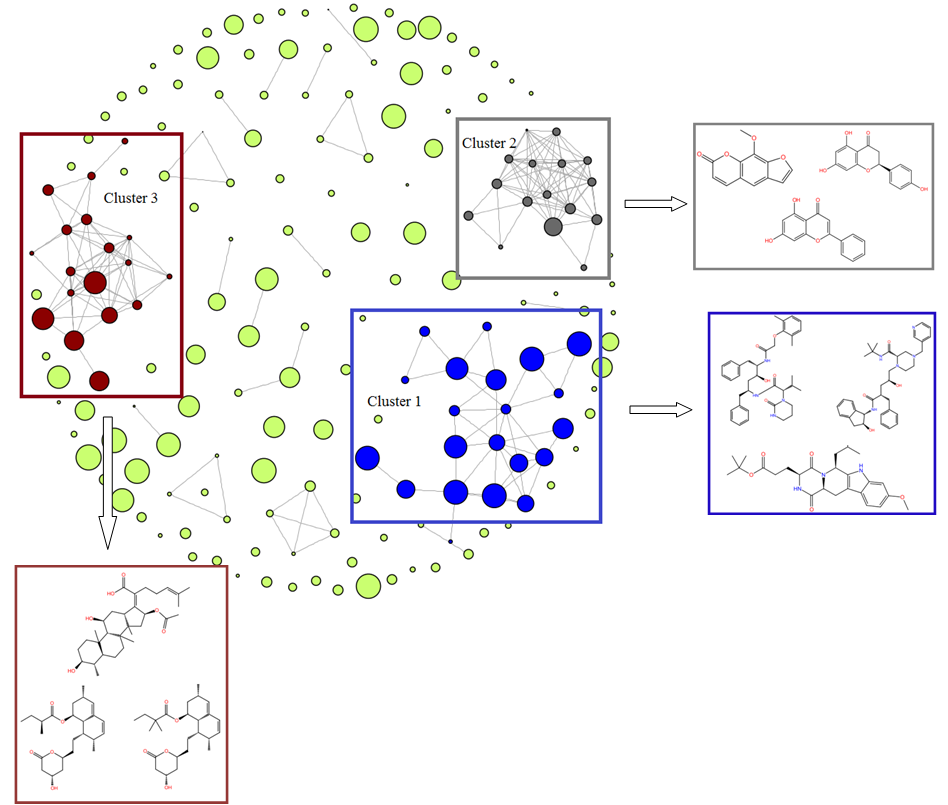


**Figure S4.**Sequence alignment of human BSEP with corrected mouse P-glycoprotein structure (PDB ID: 4M1M). The residues are colored according to the ClustalX scheme using Jalview.


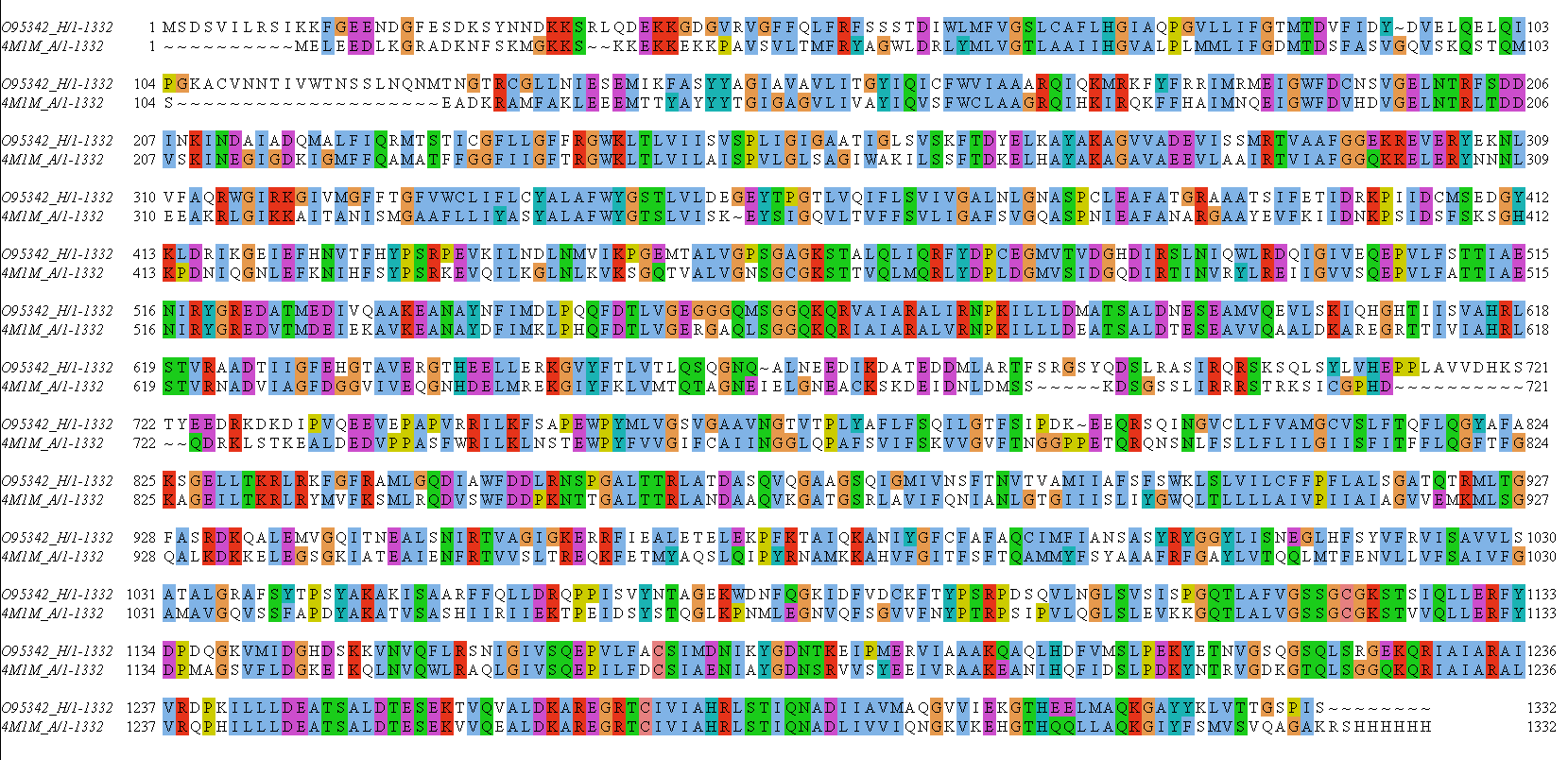


**Figure S5.** Ramachandran plot for the final homology model of human BSEP taken from PDBsum.


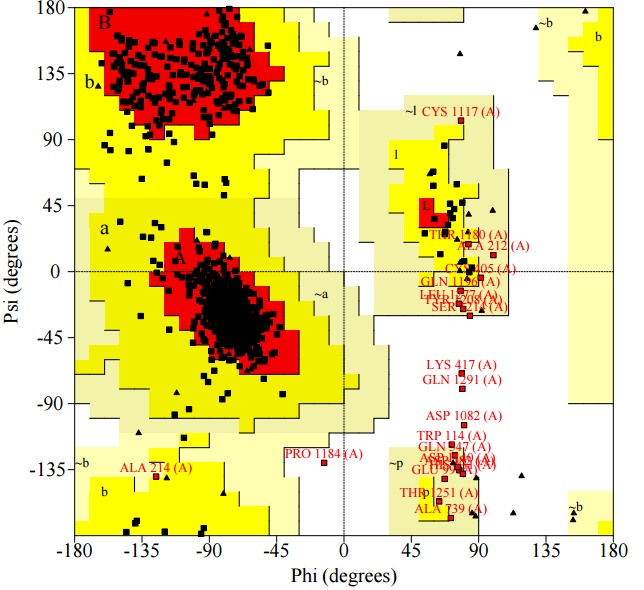


**Figure S6.** Residues that are present in the disallowed region in the final BSEP homology model.


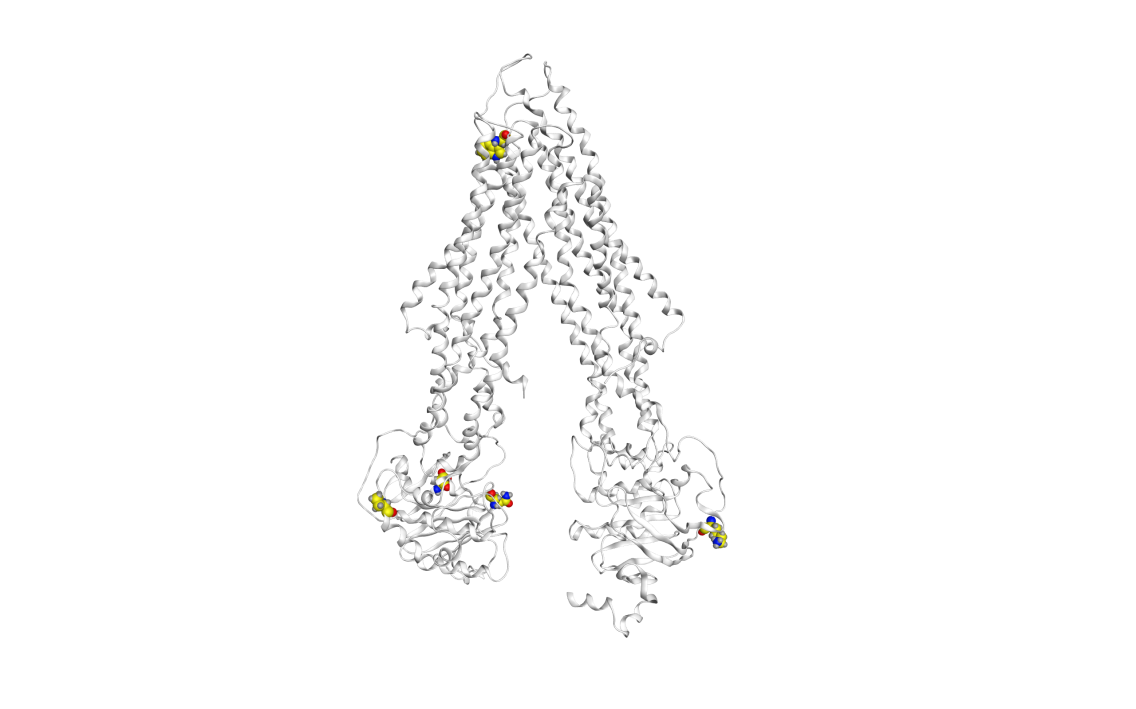


**Figure S7.** The location of Asn109, Asn116, Asn122 and Asn125 in EL1 of the BSEP homology model. The carbon atoms of the amino acids are colored in yellow for a better visibility.


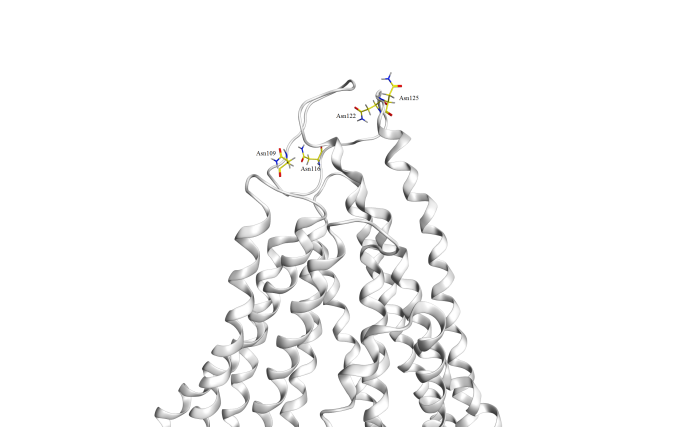


**Figure S8.**The ROC curve of ChemScore scores of training set compounds. The area under the ROC curve is 0.87.


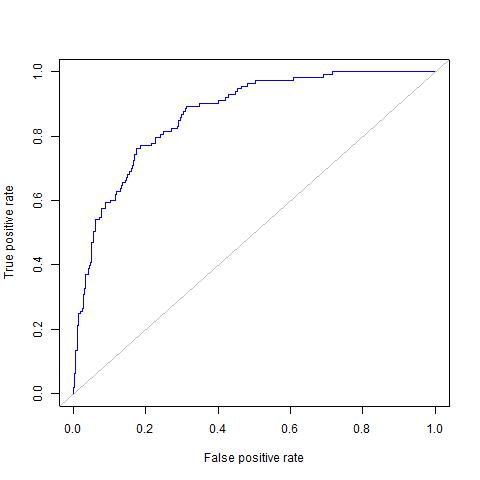


**Figure S9**. The ROC curve of GoldScore scores of training set compounds. The area under the ROC curve is 0.82.


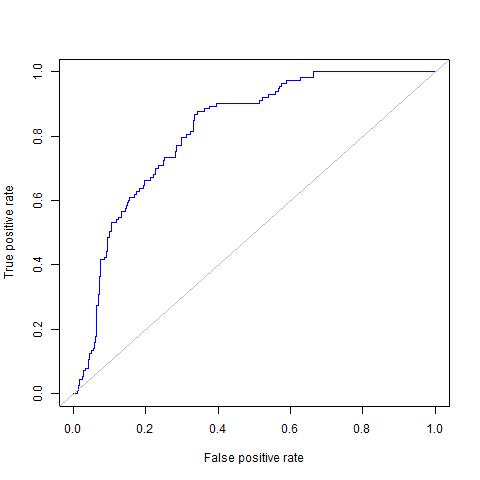


**Figure S10**. The ROC curve of GlideXP scores of training set compounds. The area under the ROC curve is 0.77.


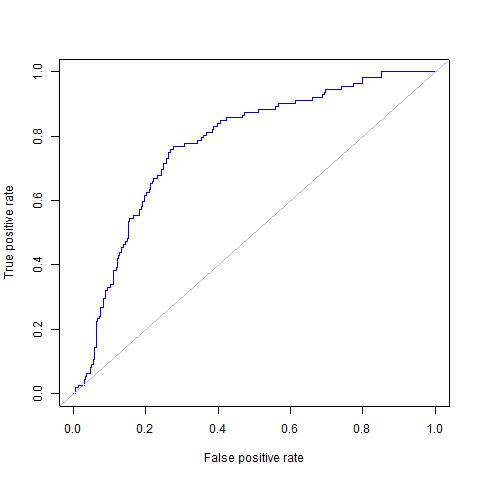


**Figure S11**. The ROC curve of Xscore(ChemScore) scores of training set compounds. The area under the ROC curve is 0.92.


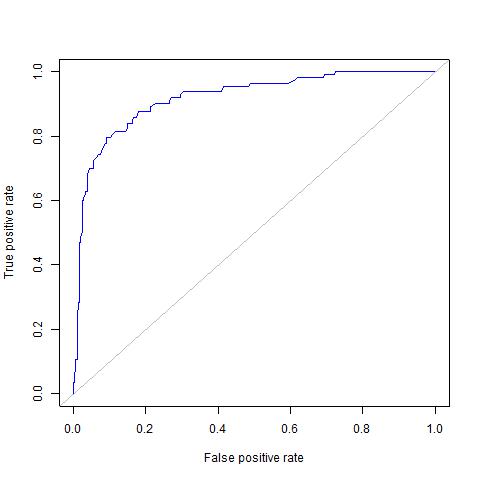


**Figure S12**. The ROC curve of Xscore(GoldScore) scores of training set compounds. The area under the ROC curve is 0.93.


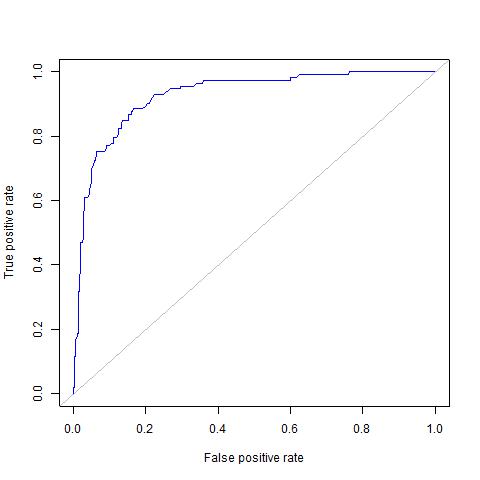


**Figure S13**. (a) Hydrophobic interaction - (b) hydrogen bond interaction fingerprints of true positives (TPs) and true negatives (TNs) of the test set (Pedersen et al.). The classification of the compounds is based on the ChemScore scoring function.

**a**


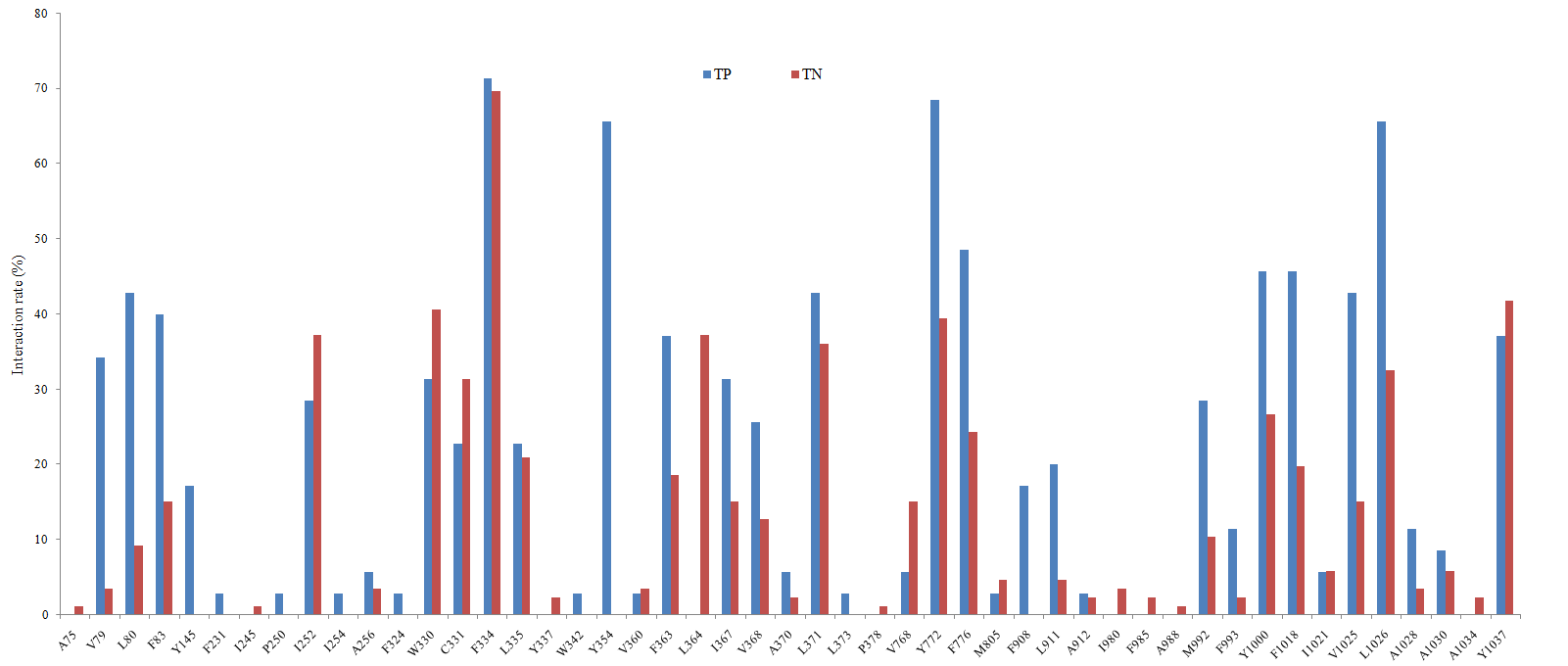


**b**


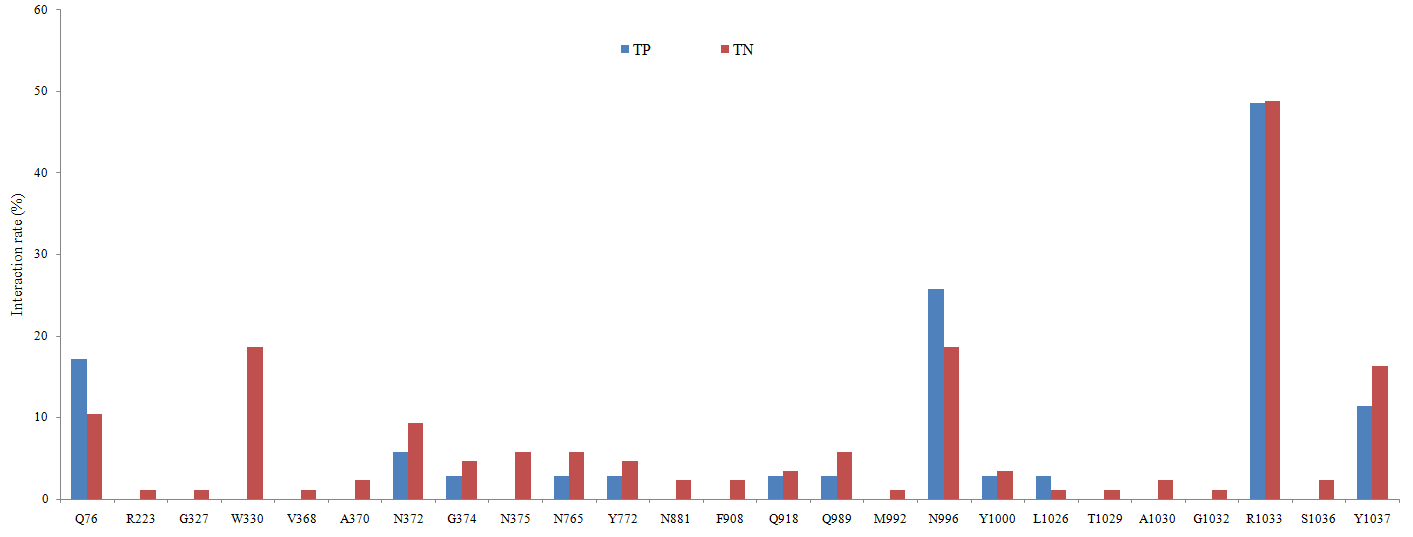


**Figure S14.** (a) Hydrophobic interaction - (b) hydrogen bond interaction fingerprints of true positives (TPs) and true negatives (TNs) of the test set (AstraZeneca-unpublished). The classification of the compounds is based on the ChemScore scoring function.

**a**


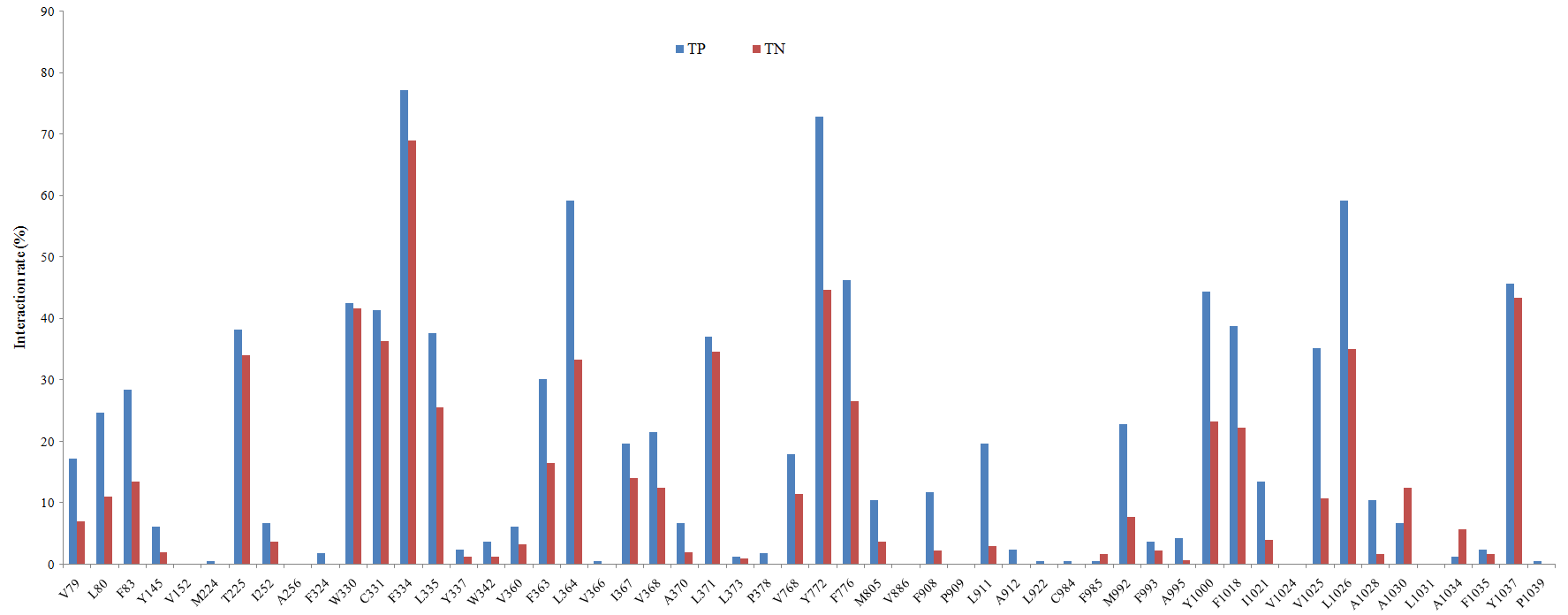


**b**


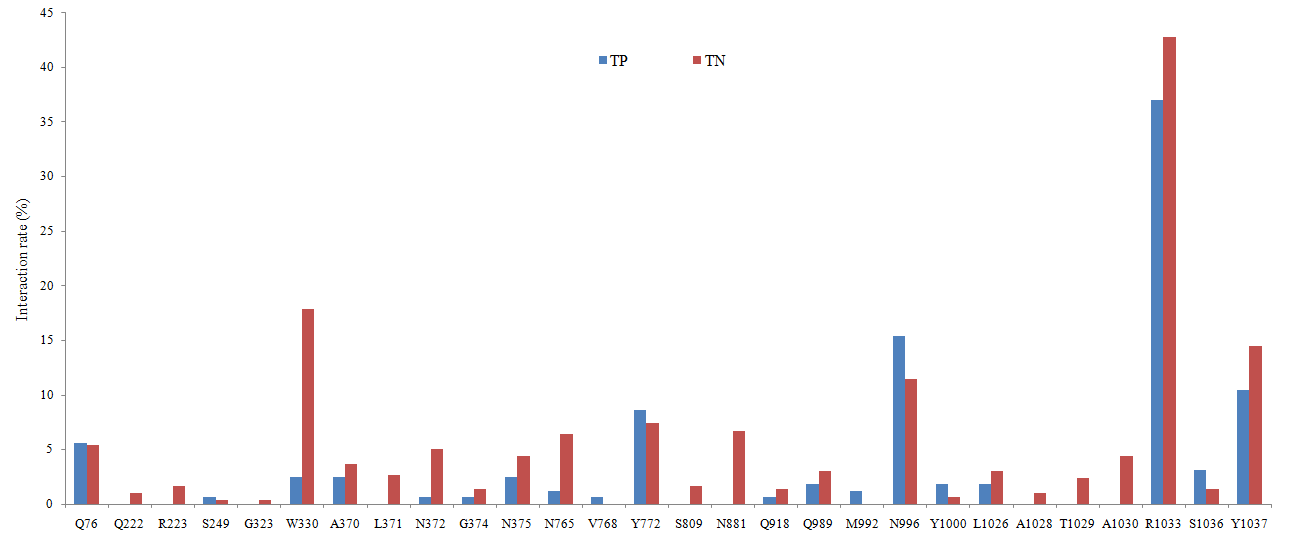


**Figure S15**. Distribution of BSEP inhibitors and non-inhibitors based on the (a) Molecular Weight (b) logP(o/w) of the test set (Pedersen et al.)

**a b
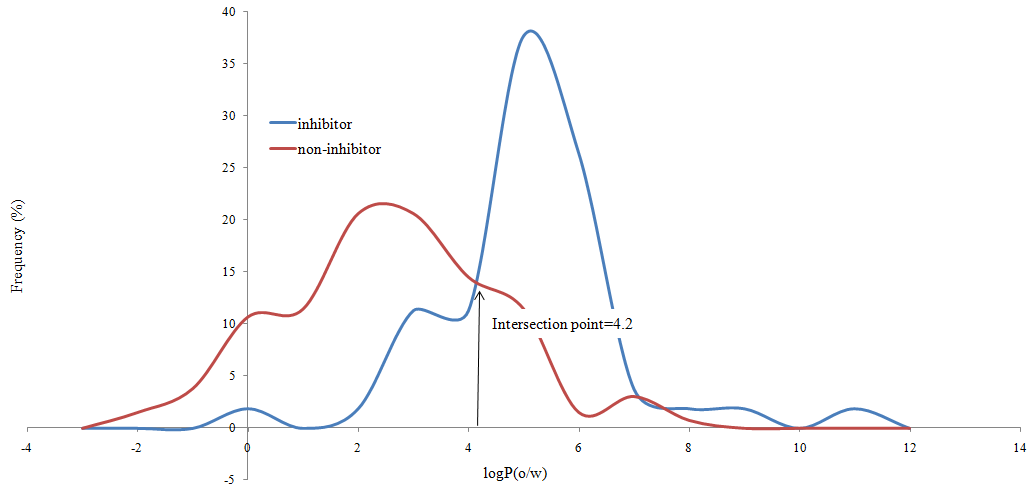
**


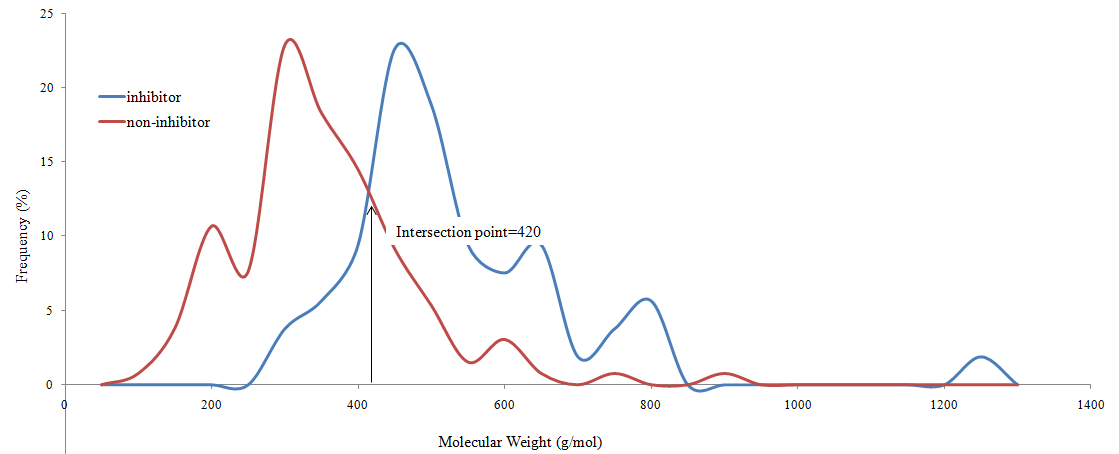


**Figure S16**. Distribution of BSEP inhibitors and non-inhibitors based on the (a) Molecular Weight (b) logP(o/w) of the test set (AstraZeneca-unpublished)

**a b
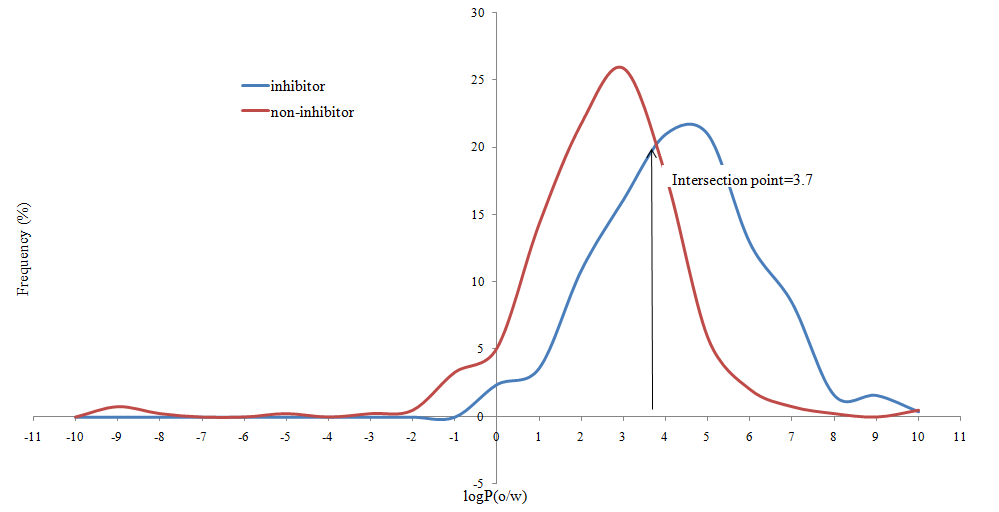
**


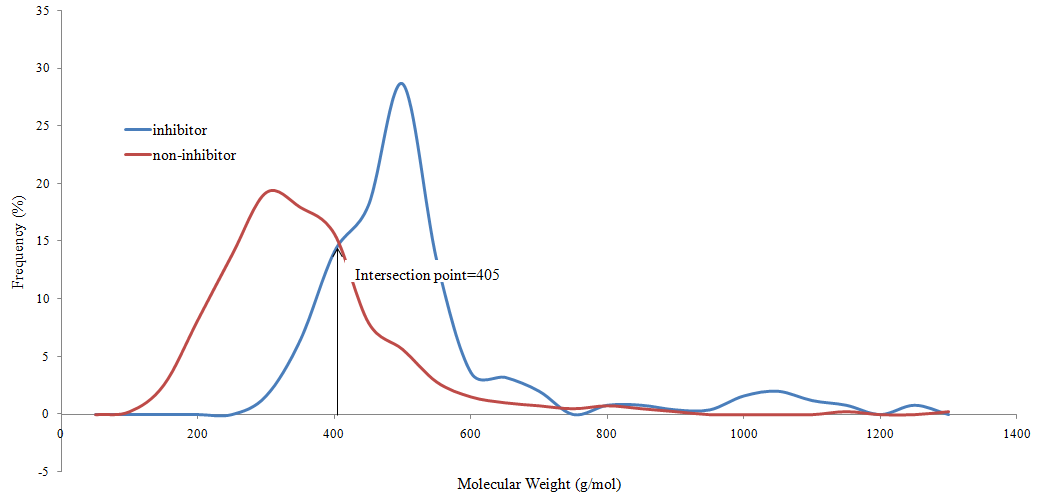


**Figure S17**. Distribution of BSEP inhibitors and non-inhibitors (training set) based on GoldScore scoring. Sensitivity, specificity, precision and MCC were calculated from the confusion matrix based on the intersection point of both curves.


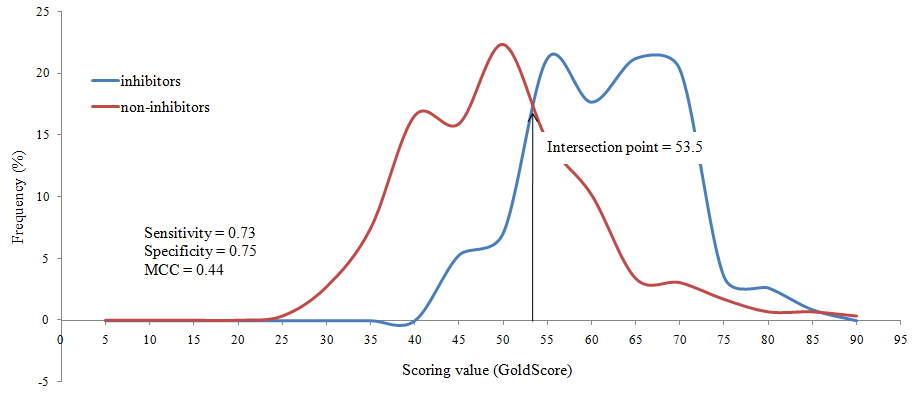


**Figure S18**. Distribution of BSEP inhibitors and non-inhibitors (training set) based on GlideXP scoring. Sensitivity, specificity, precision and MCC were calculated from the confusion matrix based on the intersection point of both curves.


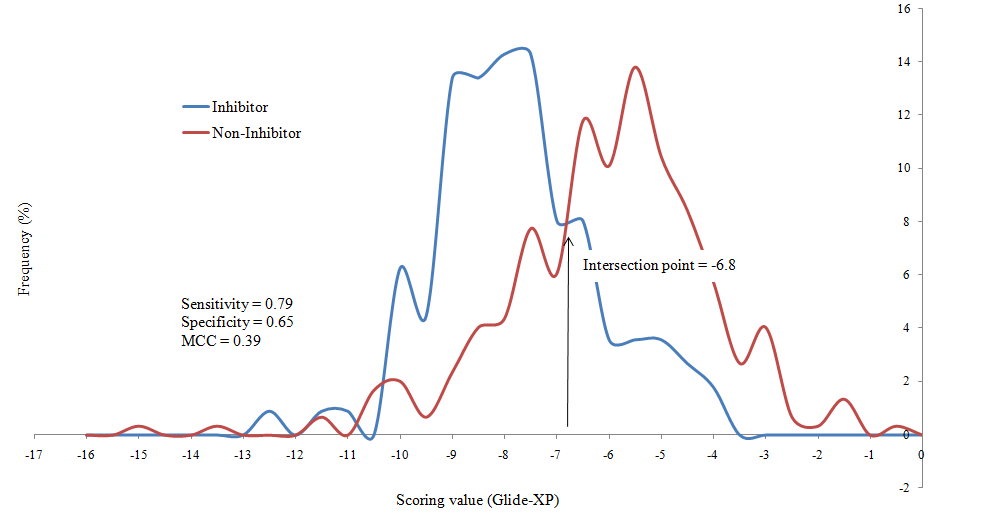


**Figure S19**. Distribution of BSEP inhibitors and non-inhibitors (training set) based on rescoring using Xscore score (poses generated using ChemScore). Sensitivity, specificity, precision and MCC were calculated from the confusion matrix based on the intersection point of both curves.


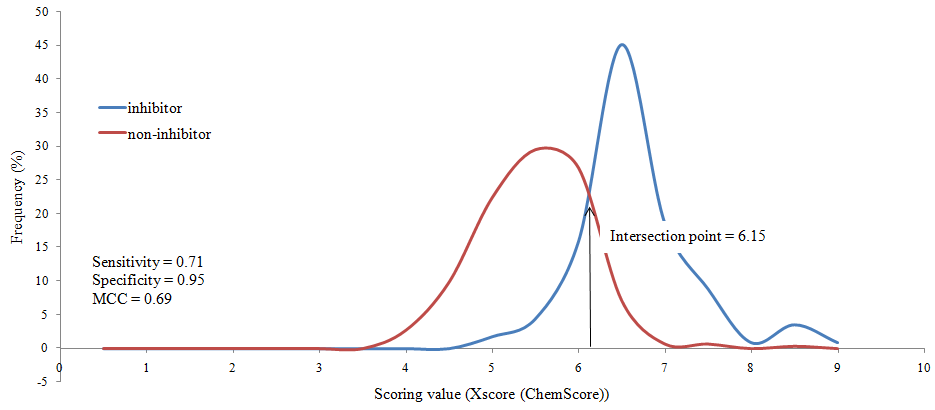


**Figure S20**. Distribution of BSEP inhibitors and non-inhibitors (training set) based on rescoring using Xscore score (poses generated using GoldScore). Sensitivity, specificity, precision and MCC were calculated from the confusion matrix based on the intersection point of both curves.


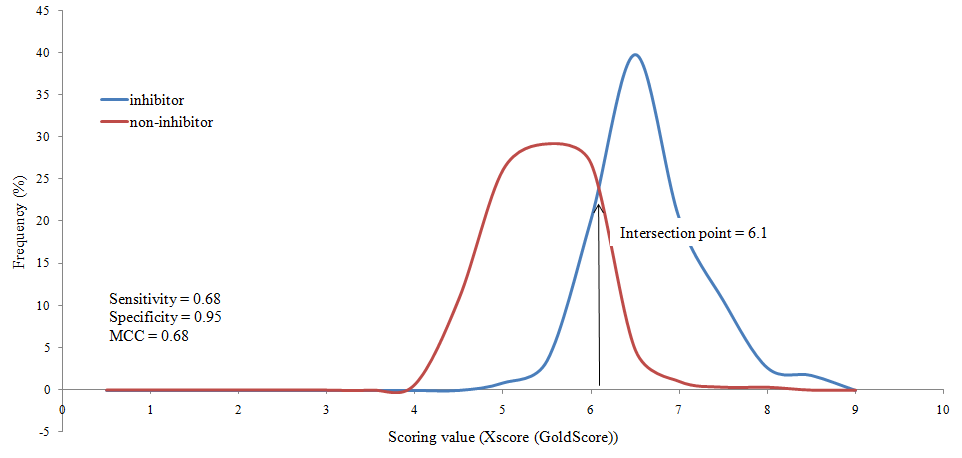


**Figure S21**. Distribution of BSEP inhibitors and non-inhibitors (training set) based on ChemScore scoring and molecular weight. Sensitivity, specificity, precision and MCC were calculated from the confusion matrix based on the intersection point of both curves.


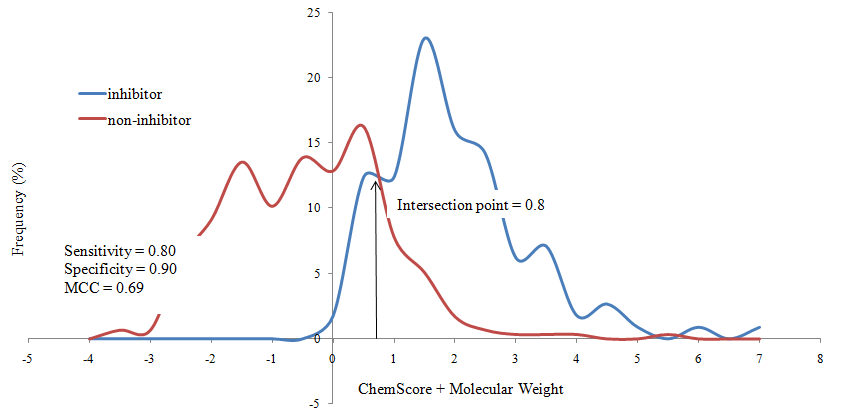


**Figure S22.** Distribution of BSEP inhibitors and non-inhibitors (training set) based on ChemScore scoring and logP. Sensitivity, specificity, precision and MCC were calculated from the confusion matrix based on the intersection point of both curves.


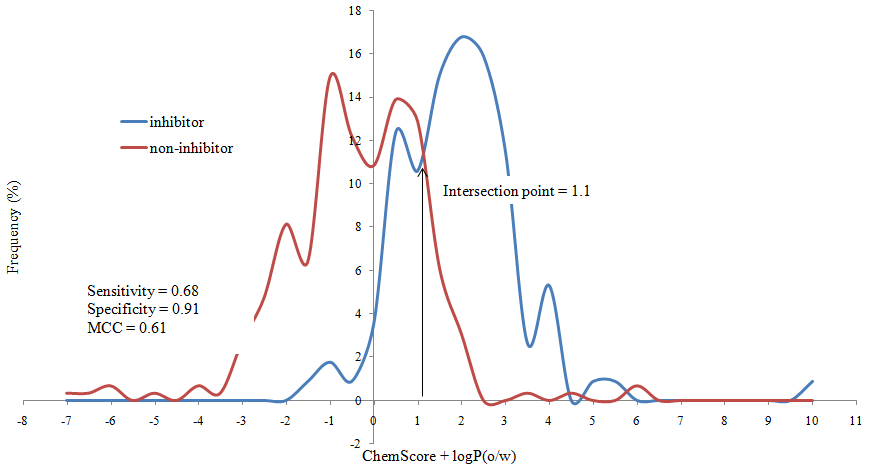


**Figure S23**. Distribution of BSEP inhibitors and non-inhibitors (training set) based on ChemScore scoring and Molecular Weight and logP. Sensitivity, specificity, precision and MCC were calculated from the confusion matrix based on the intersection point of both curves.


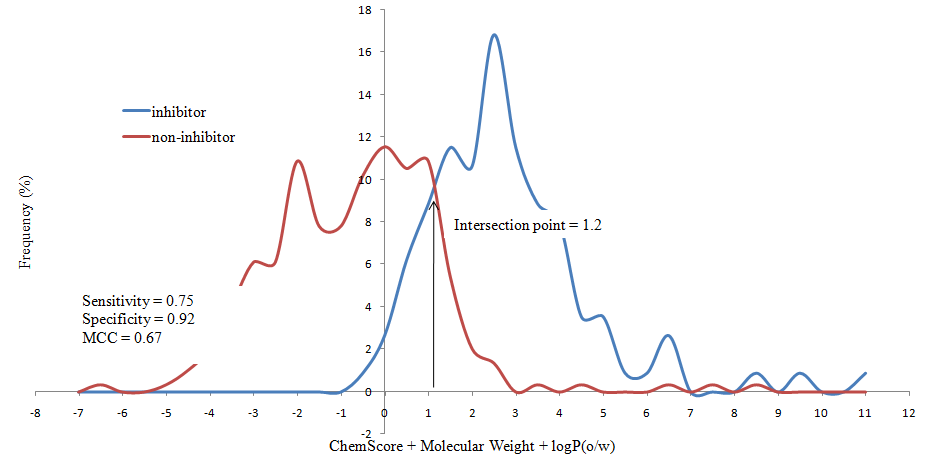


**Figure S24**. Distribution of BSEP inhibitors and non-inhibitors (training set) based on GoldScore rescoring and Molecular Weight. Sensitivity, specificity, precision and MCC were calculated from the confusion matrix based on the intersection point of both curves.


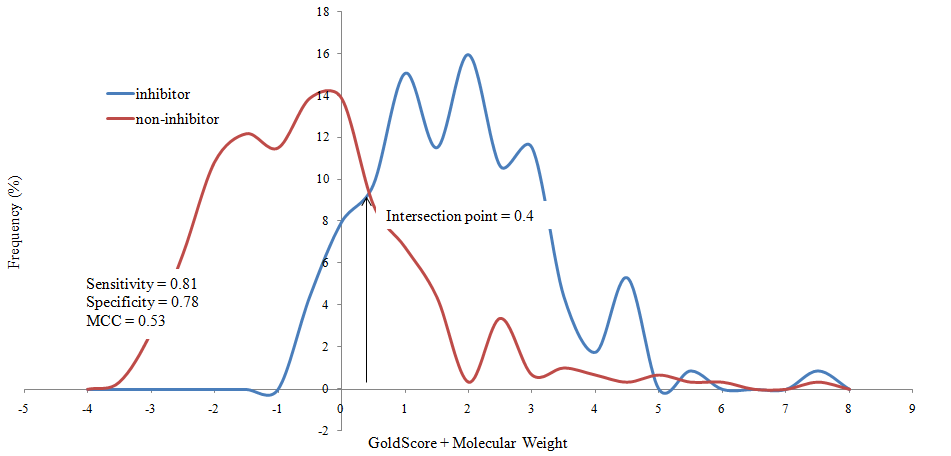


**Figure S25**. Distribution of BSEP inhibitors and non-inhibitors (training set) based on GoldScore scoring and logP. Sensitivity, specificity, precision and MCC were calculated from the confusion matrix based on the intersection point of both curves.


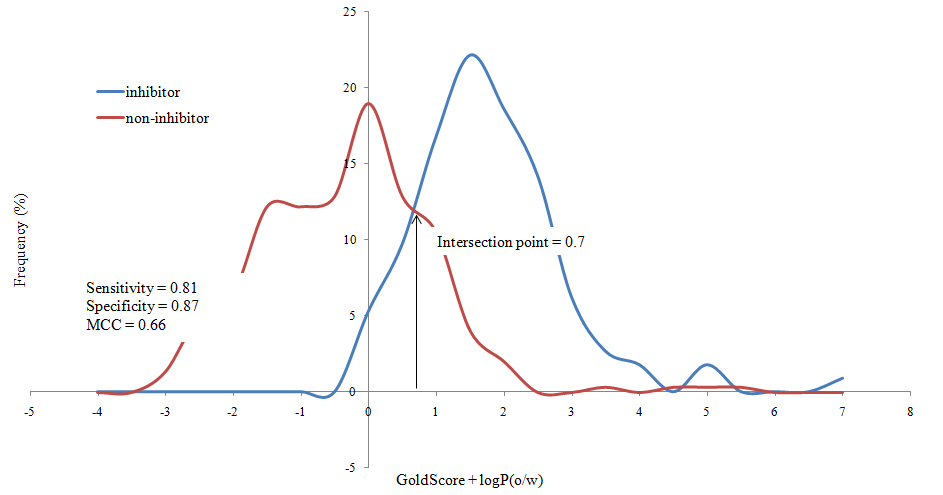


**Figure S26**. Distribution of BSEP inhibitors and non-inhibitors (training set) based on GoldScore scoring and Molecular Weight and logP. Sensitivity, specificity, precision and MCC were calculated from the confusion matrix based on the intersection point of both curves.


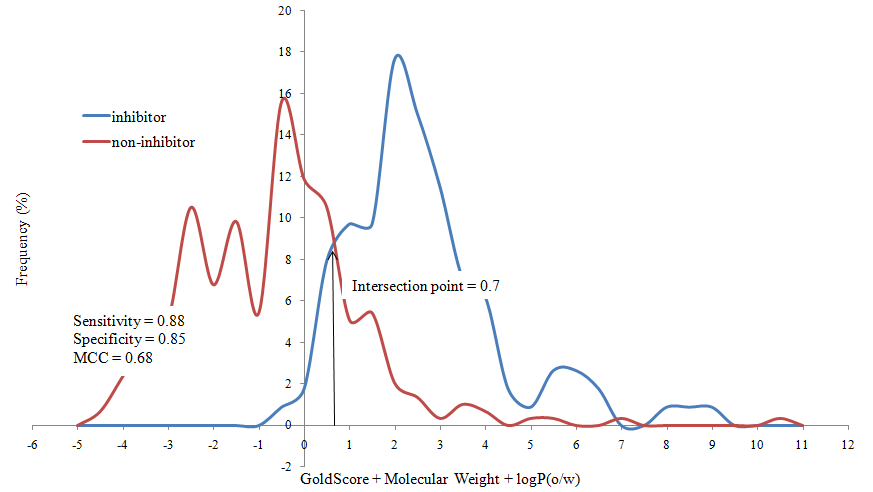


**Figure S27**. Distribution of BSEP inhibitors and non-inhibitors (training set) based on Xscore (ChemScore) and Molecular Weight. Sensitivity, specificity, precision and MCC were calculated from the confusion matrix based on the intersection point of both curves.


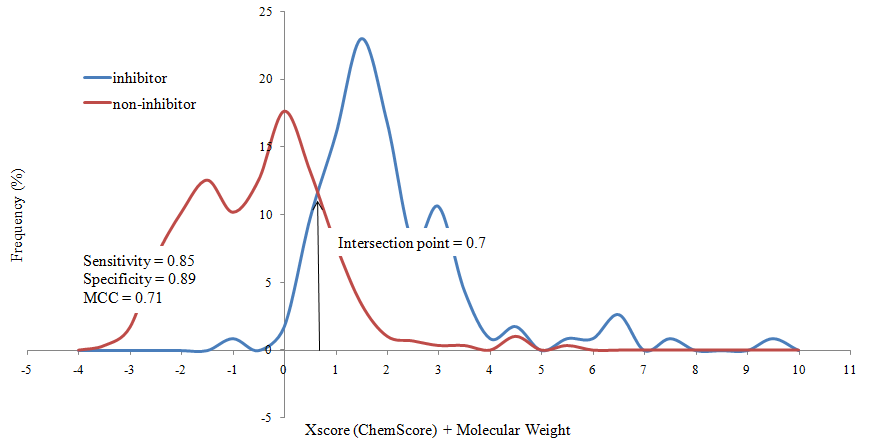


**Figure S28**. Distribution of BSEP inhibitors and non-inhibitors (training set) based on Xscore (ChemScore) and logP. Sensitivity, specificity, precision and MCC were calculated from the confusion matrix based on the intersection point of both curves.


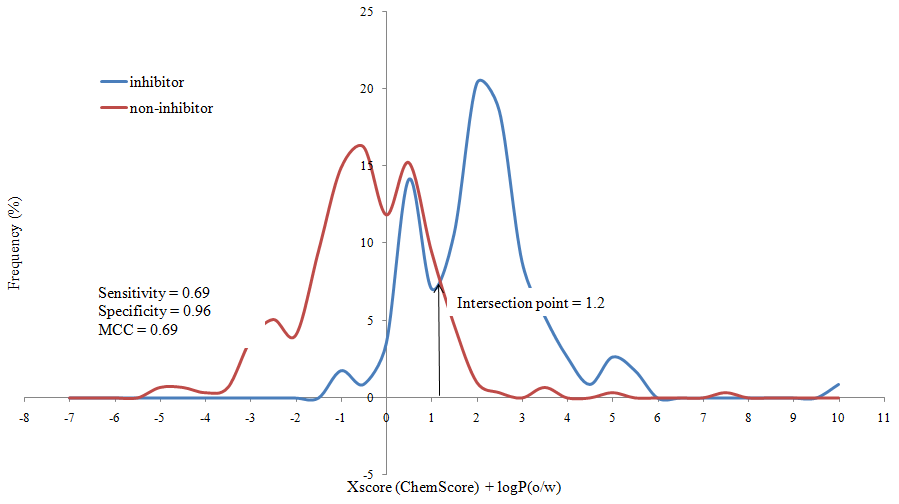


**Figure S29.** Distribution of BSEP inhibitors and non-inhibitors (training set) based on Xscore (ChemScore) and Molecular Weight and logP. Sensitivity, specificity, precision and MCC were calculated from the confusion matrix based on the intersection point of both curves.


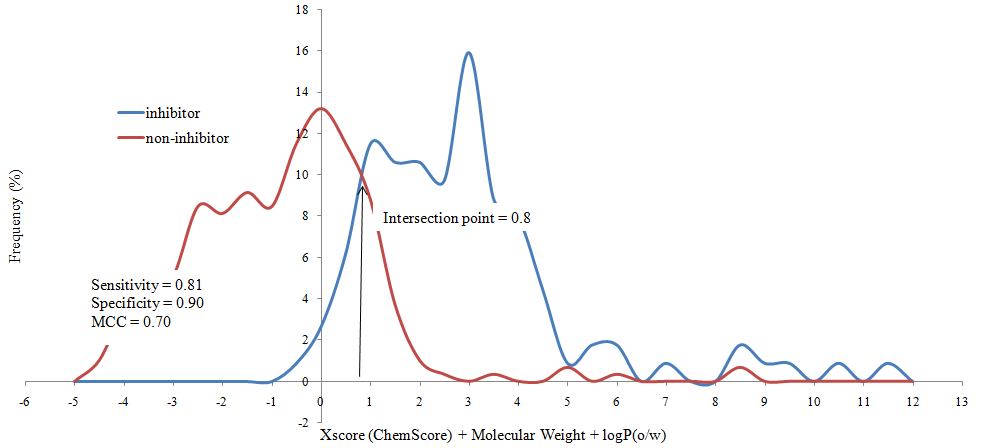


**Figure S30.** Distribution of BSEP inhibitors and non-inhibitors (training set) based on Xscore (GoldScore) and Molecular Weight. Sensitivity, specificity, precision and MCC were calculated from the confusion matrix based on the intersection point of both curves.


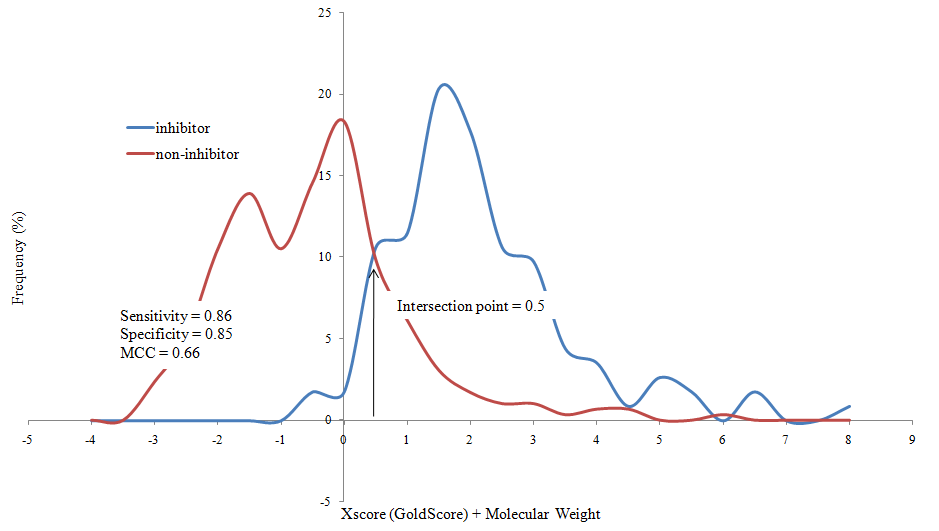


**Figure S31.** Distribution of BSEP inhibitors and non-inhibitors (training set) based on Xscore (GoldScore) and logP. Sensitivity, specificity, precision and MCC were calculated from the confusion matrix based on the intersection point of both curves.


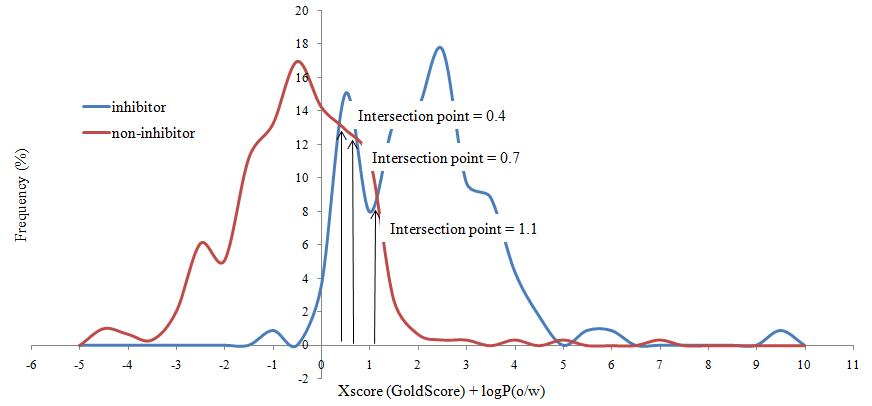


**Figure S32.** Distribution of BSEP inhibitors and non-inhibitors (training set) based on Xscore (GoldScore) and Molecular Weight and logP. Sensitivity, specificity, precision and MCC were calculated from the confusion matrix based on the intersection point of both curves.


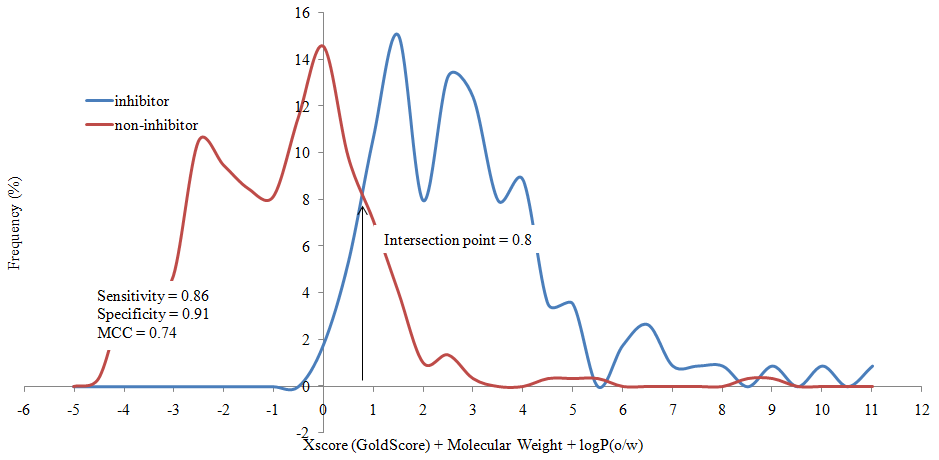


**Figure S33.** Distribution of functional groups in the test set (a) Pedersen et al. (b) AstraZeneca (unpublished) dataset classified using ChemScore rescoring function.

**a**


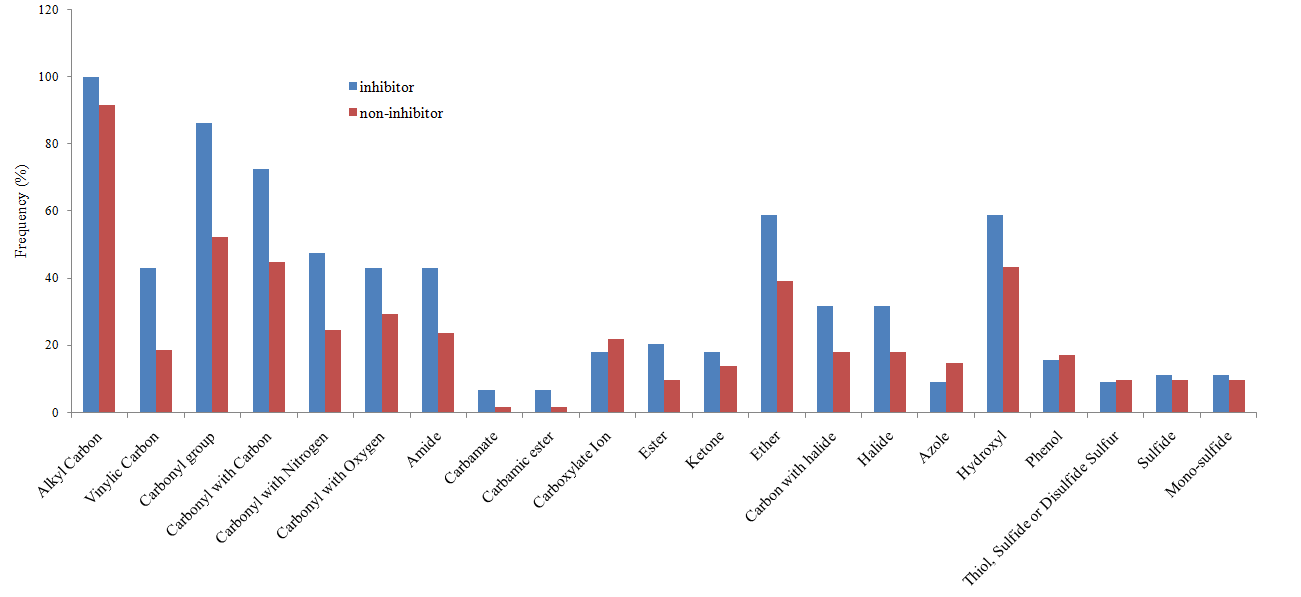


**b**


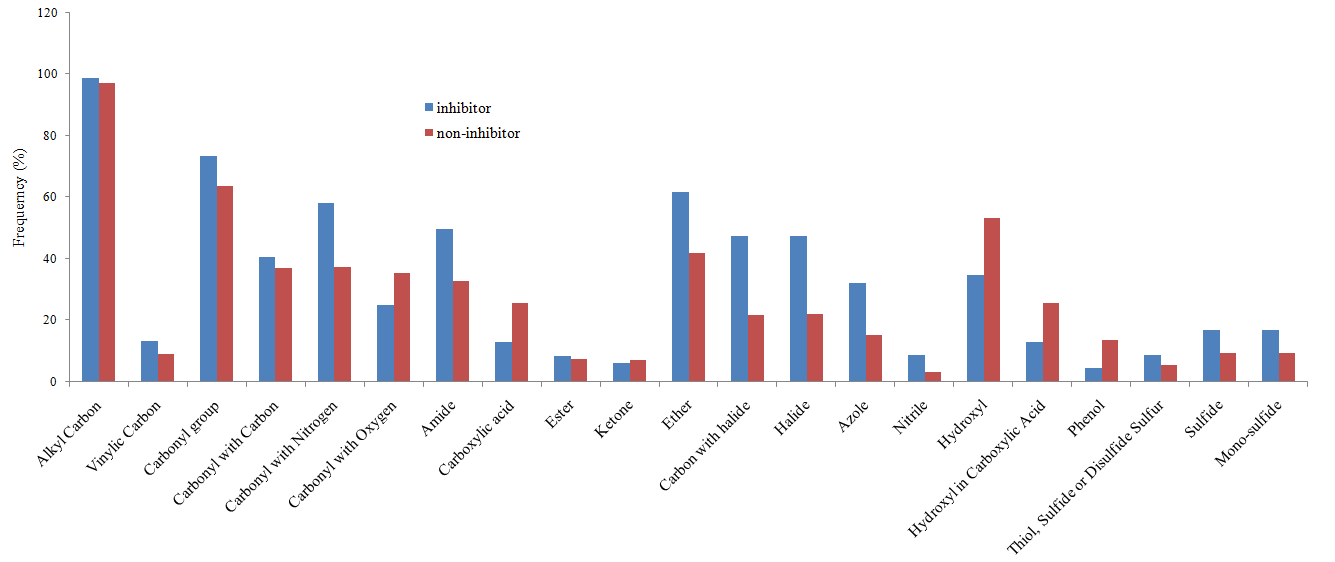


**Figure S34.**Heat map from PLIF analysis for training set non-inhibitors (x-axis: contact residues; y-axis: functional groups in the ligand showing interaction with the residue; color scale: number of interacting ligands).


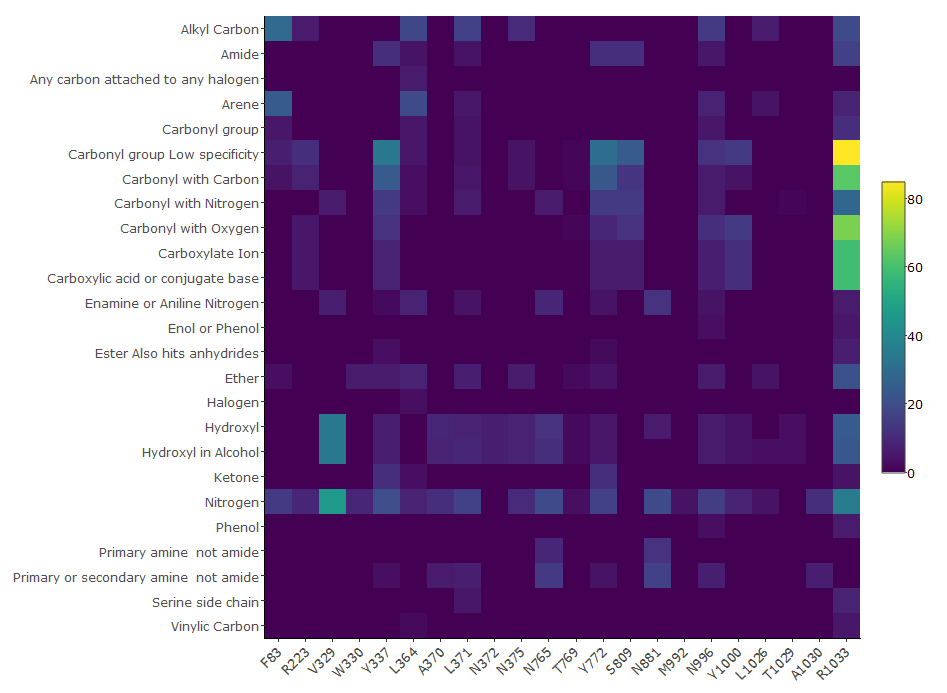


**Figure S35.**Heat map from PLIF analysis for test set (a) inhibitors (b) non-inhibitors (Pedersen et al.) (x-axis: contact residues; y-axis: functional groups in the ligand showing interaction with the residue; color scale: number of interacting ligands).

**a**


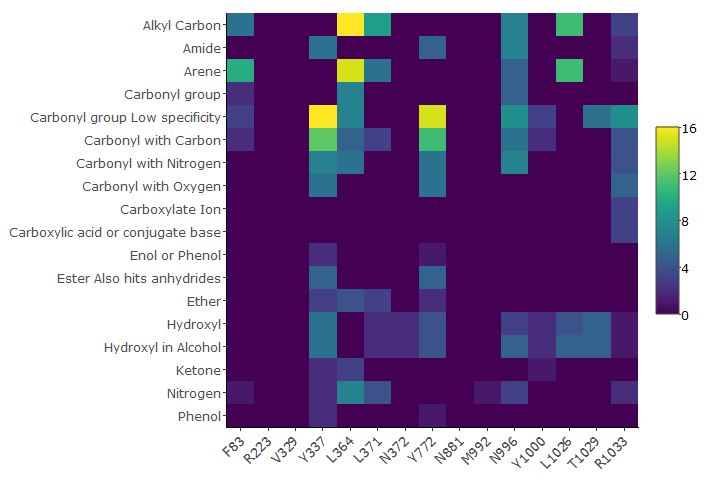


**b**


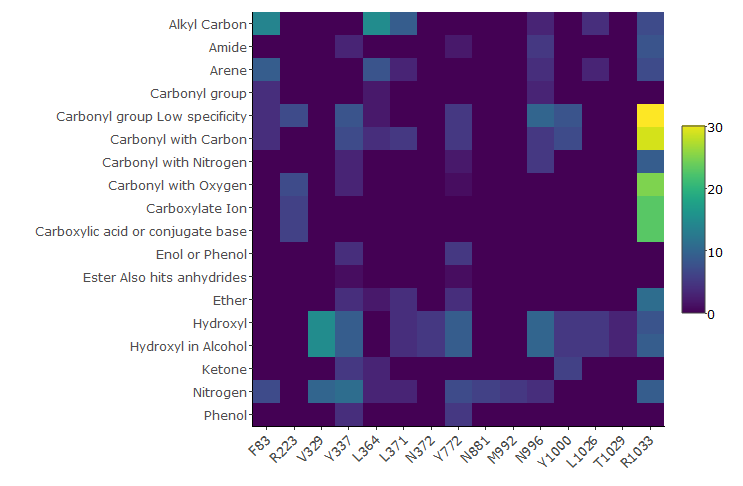


**Figure S36.**Heat map from PLIF analysis for test set (a) inhibitors (b) non-inhibitors (AstraZeneca-unpublished) (x-axis: contact residues; y-axis: functional groups in the ligand showing interaction with the residue; color scale: number of interacting ligands).

**a**


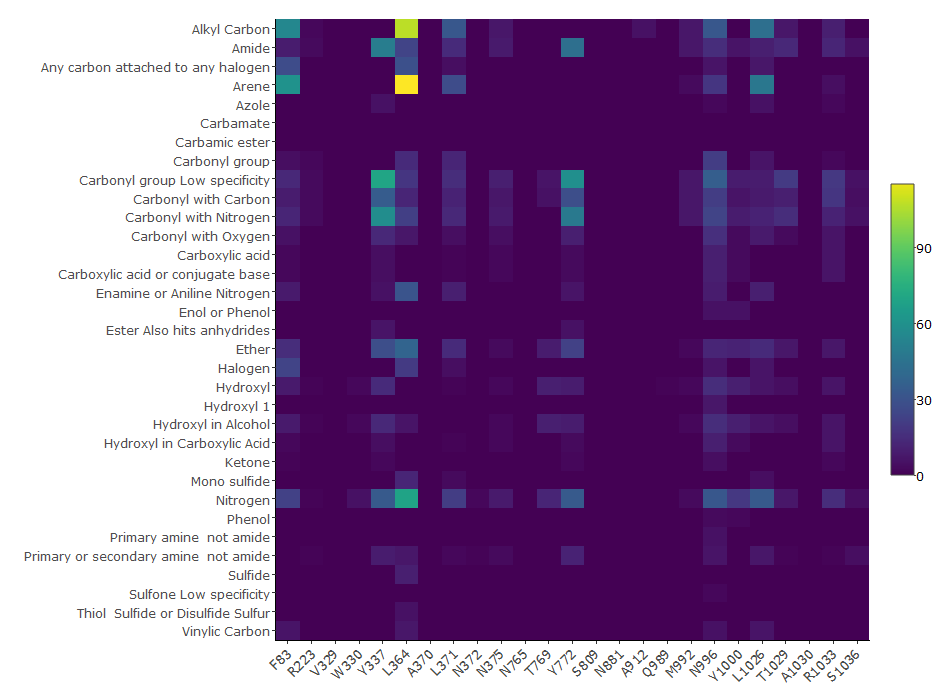


**b**


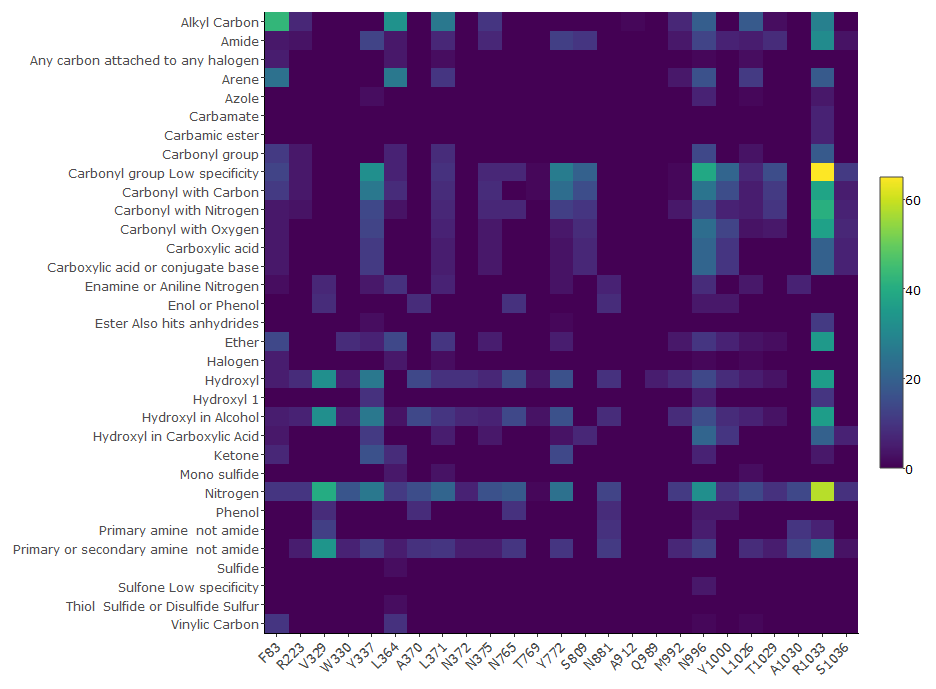


**Classification using Protein Ligand Interaction Fingerprints (PLIF)**

Finally, we wanted to assess a measure of PLIF homogeneity within the inhibitors of the training set. Therefore, we calculated the Tanimoto coefficients for each inhibitor versus the remaining inhibitors on basis of their PLIFs, and finally averaged the resulting coefficients. The averaged PLIF Tanimoto coefficient describes an inhibitor’s PLIF similarity in relation to all inhibitors. The same procedure was undertaken for all inhibitors in the training set. From the distribution of averaged coefficients we calculated the mean and the standard deviation. Finally, a critical value was defined by subtraction of the standard deviation from the mean. This critical value was used as a threshold to classify compounds as inhibitors or non-inhibitors from the test dataset. To classify a test compound with this approach, the PLIF vector of the compound is used to calculate Tanimoto similarites against all compounds of the inhibitors in the training set. After averaging the calculated coefficients of the test compound, the resulting mean is compared against the critical value. If the averaged Tanimoto coefficients of the test compound is greater than the critical value, it is classified as an inhibitor, otherwise as a non-inhibitor. The PLIF-based classification provided accuracy measures comparable to those obtained from the docking score based classification (Table S5 in the supplementary material).

Moreover, information obtained using PLIF analysis in a sequential fashion i.e. reassessment of true positives and false positives obtained via the docking score based classification using PLIF-based similarity, improved the classification precision for both the training and the external test datasets (Table S5 in the supplementary material). The highest precision was obtained using the third PLIF approach that encoded residues along with the functional groups of the interacting ligand. Using this method, we achieved a precision of 0.87 (accuracy = 84%) for the training set. The same model showed a precision of 0.72 for the test dataset from Pedersen et al. [34] (accuracy = 84%) and 0.79 for the test dataset from AstraZeneca (accuracy = 76%). Overall, the number of false positives could be significantly reduced using the PLIF based classification.
